# Small mitochondrial protein NERCLIN regulates cardiolipin homeostasis and mitochondrial ultrastructure

**DOI:** 10.1073/pnas.2210599120

**Published:** 2023-07-18

**Authors:** Svetlana Konovalova, Rubén Torregrosa-Muñumer, Pooja Manjunath, Xiaonan Liu, Sundar Baral, Kaneez Fatima, Minna Holopainen, Jouni Kvist, Jayasimman Rajendran, Yang Yang, Markku Varjosalo, Reijo Käkelä, Pentti Somerharju, Henna Tyynismaa

**Affiliations:** ^a^Stem Cells and Metabolism Research Program, Research Programs Unit, Faculty of Medicine, University of Helsinki, 00014 Helsinki, Finland; ^b^Institute of Biotechnology, Helsinki Institute of Life Science, University of Helsinki, 00014 Helsinki, Finland; ^c^Molecular and Integrative Biosciences Research Program, Faculty of Biological and Environmental Sciences, University of Helsinki, 00014 Helsinki, Finland; ^d^Helsinki University Lipidomics Unit, Helsinki Institute of Life Science and Biocenter Finland, University of Helsinki, 00014 Helsinki, Finland; ^e^School of Life and Health Sciences, Faculty of Science, The Chinese University of Hong Kong, 518172 Shenzhen, China; ^f^Department of Biochemistry and Developmental Biology, Faculty of Medicine, University of Helsinki, 00014 Helsinki, Finland

**Keywords:** NERCLIN, cardiolipin, OPA1, prohibitins, small mitochondrial proteins

## Abstract

Cardiolipin (CL) is an essential phospholipid for mitochondrial structure and function. Here, we present a small mitochondrial protein, NERCLIN, as a negative regulator of CL homeostasis and mitochondrial ultrastructure. Primate-specific NERCLIN is expressed ubiquitously from the *GRPEL2* locus on a tightly regulated low level. NERCLIN overexpression severely disrupts mitochondrial cristae structure and induces mitochondrial fragmentation. Proximity labeling and immunoprecipitation analysis suggested interactions of NERCLIN with CL synthesis and prohibitin complexes on the matrix side of the inner mitochondrial membrane. Lipid analysis indicated that NERCLIN regulates mitochondrial CL content. Furthermore, NERCLIN is responsive to heat stress ensuring OPA1 processing and cell survival. Thus, we propose that NERCLIN contributes to the stress-induced adaptation of mitochondrial dynamics. Our findings add NERCLIN to the group of recently identified small mitochondrial proteins with important regulatory functions.

## Significance

Recent studies have shown that the human mitochondrial proteome is enriched for small proteins with vital cellular functions. We describe here a primate-specific small mitochondrial protein NERCLIN as a negative regulator of cardiolipin homeostasis and mitochondrial ultrastructure. NERCLIN has a protective role in heat stress, contributing to the stress-induced adaption of mitochondrial dynamics. Our findings add NERCLIN to the group of small mitochondrial proteins with important regulatory functions.

Alternative splicing is one of the main sources of protein diversity in higher eukaryotes (1). Although high-throughput techniques routinely detect thousands of alternatively spliced transcripts, proteomics analysis often fails to identify a vast majority of them. Short isoforms of canonical proteins are particularly difficult to detect since the existing algorithms usually exclude them from the proteome annotation. Several recent studies have identified small proteins that play important roles in fundamental biological processes such as muscle development and activity (2–5), mRNA degradation (6), and transcriptional repression (7, 8). Interestingly, the mitochondrial proteome is particularly enriched for small proteins (9), many of which have vital cellular functions. For example, BRAWNIN, a 71-amino-acid peptide encoded by *C12orf73*, is essential for vertebrate oxidative phosphorylation (9), a 54-amino-acid mitochondrial microprotein PIGBOS regulates the unfolded protein response (10), and a 56-amino-acid microprotein MTLN (mitoregulin) controls mitochondrial protein complex assembly (11) and adipocyte metabolism (12).

Cardiolipin (CL) is a mitochondria-specific phospholipid, which is essential for functional shaping of the mitochondrial inner membrane, thus contributing to cristae structure and stabilization of respiratory chain complexes and other components important for bioenergetics (13, 14). CL is synthesized within the inner membrane by the consecutive actions of phosphatidylglycerophosphate synthase (PGS1), phosphatidylglycerophosphate phosphatase (PTPMT1), and CL synthase (CLS1), followed by fatty acyl chain remodeling to generate mature CL species (15). PGS1, PTPMT1, and CLS1 form a CL synthesis complex, which interacts with cristae membrane-organizing proteins such as prohibitins and stomatin-like protein 2 (STML2) (16, 17). Abnormal CL content or species composition disrupts mitochondrial function and form and is linked to mitophagy, the selective mitochondrial degradation pathway (14, 15, 18, 19).

Here, we describe the identification of NERCLIN, a human mitochondrial protein of 90 amino acids in its mature form, which is produced as an alternative splice variant of the *GRPEL2* gene. GRPEL2 is a nucleotide exchange factor for mitochondrial protein import chaperone mtHSP70 (20, 21), however, NERCLIN does not share the amino acids required for the canonical function of GRPEL2. Instead, our results suggest that NERCLIN regulates CL metabolism and mitochondrial morphology.

## Results

### Identification of NERCLIN, a Splice Variant of Human *GRPEL2*.

**Fig. 1.**
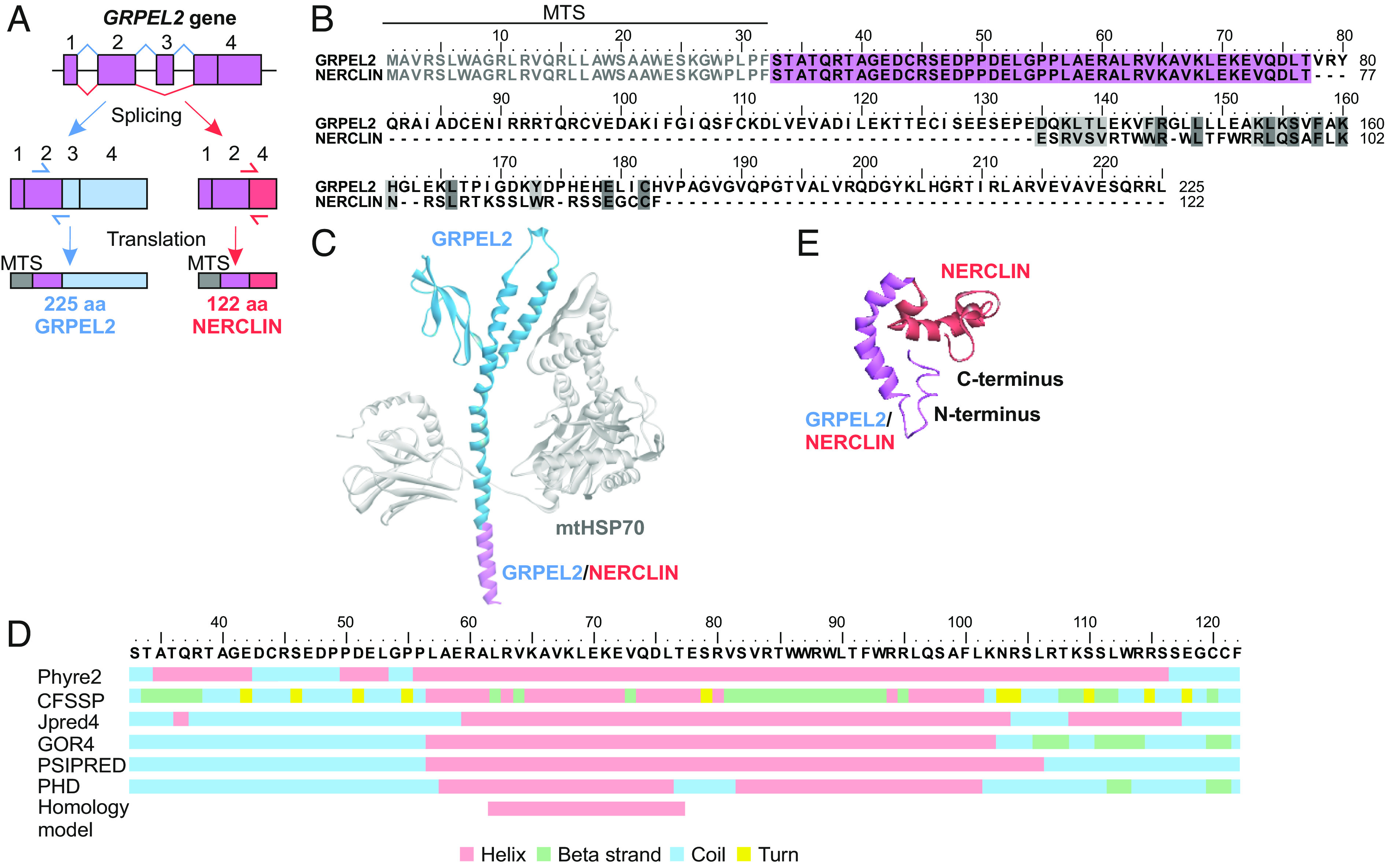
Identification of NERCLIN, a splice variant of human *GRPEL2*. (*A*) Schematic representation of the alternative splicing of the human *GRPEL2* gene, highlighting the parts of the transcripts and proteins that are specific for *GRPEL2* in blue and for *NERCLIN* in red. The mitochondrial targeting sequence (MTS) is in gray, followed by the part of the protein common for GRPEL2 and NERCLIN in violet. The location of the transcript-specific primer pairs is shown by arrows. (*B*) Amino acid sequence alignment of human GRPEL2 and NERCLIN. Identical residues are highlighted in dark gray; similar residues are in light gray. The amino acid sequence common for GRPEL2 and NERCLIN is in violet. MTS (1 to 32 amino acids) is in gray. (*C*) Structural model of GRPEL2 interacting with mtHSP70 (gray). The N-terminal α-helix present in NERCLIN is in violet; the rest of the GRPEL2 molecule is in blue. For simplicity, the structure of only one GRPEL2 molecule in the GRPEL2 dimer is shown. (*D*) Secondary structural prediction of human NERCLIN. For the analysis, MTS was removed from the protein sequence. (*E*) Tertiary structure prediction of human NERCLIN by SSpro server. The N-terminal α-helix present in NERCLIN is in violet, and the unique amino acid sequence for NERCLIN is in red. MTS was removed from the protein sequence.

While exploring the human *GRPEL2* gene on the UCSC Genome Browser (22) for our previous study (21), we noticed the presence of expressed sequence tags for two separate transcripts. Transcript variant one contains four exons, encoding the 225-amino-acid GRPEL2 protein, which has a 32-amino-acid N-terminal mitochondrial targeting sequence (MTS). The rare transcript variant 2 skips exon 3 producing an exon 2/4 splice junction (Fig. 1*A*). The absence of exon 3 results in the out-of-frame deletion and translational frameshift that potentially generates a 122-amino-acid protein, which we termed NERCLIN (negative regulator of CL) (Fig. 1*B*). Thus, NERCLIN shares the N-terminal MTS and successive 45 amino acids with GRPEL2 but has a unique C terminus of 45 amino acids, forming a mature protein of 90 amino acids.

We have previously generated a homology model, which shows that the C terminus of human GRPEL2 interacts with the catalytic ATPase domain of the mtHSP70 chaperone (21). As NERCLIN lacks the critical part for binding mtHSP70, it cannot perform a GrpE-like nucleotide exchange function. Instead, according to the homology model, NERCLIN corresponds to the N-terminal alpha-helix of GRPEL2 (Fig. 1*C*). Structure predictions by several algorithms support that mature NERCLIN is mainly a small alpha-helix (Fig. 1 *D* and *E*). In addition, using Phobius, TMHMM, TMFoldWeb, and, DAS algorithms, we predicted that NERCLIN does not contain transmembrane domains.

To verify that the *NERCLIN* transcript is expressed in human cells, we performed RT-PCR amplification of RNA from 143B osteosarcoma cells using primers located in *GRPEL2* exons 1 and 4. We observed two amplification products corresponding exactly to the expected amplicons from *GRPEL2* and from *NERCLIN* lacking exon 3 (*SI Appendix*, Fig. S1*A*). Nucleotide sequencing of each product confirmed that the larger transcript corresponded to *GRPEL2,* while the smaller transcript was an alternatively spliced *NERCLIN* that contained an out-of-frame deletion of the entire exon 3 comprising 82 bases (nt 232 to 314) (*SI Appendix*, Fig. S1 *A* and *B*).

**Fig. 2.**
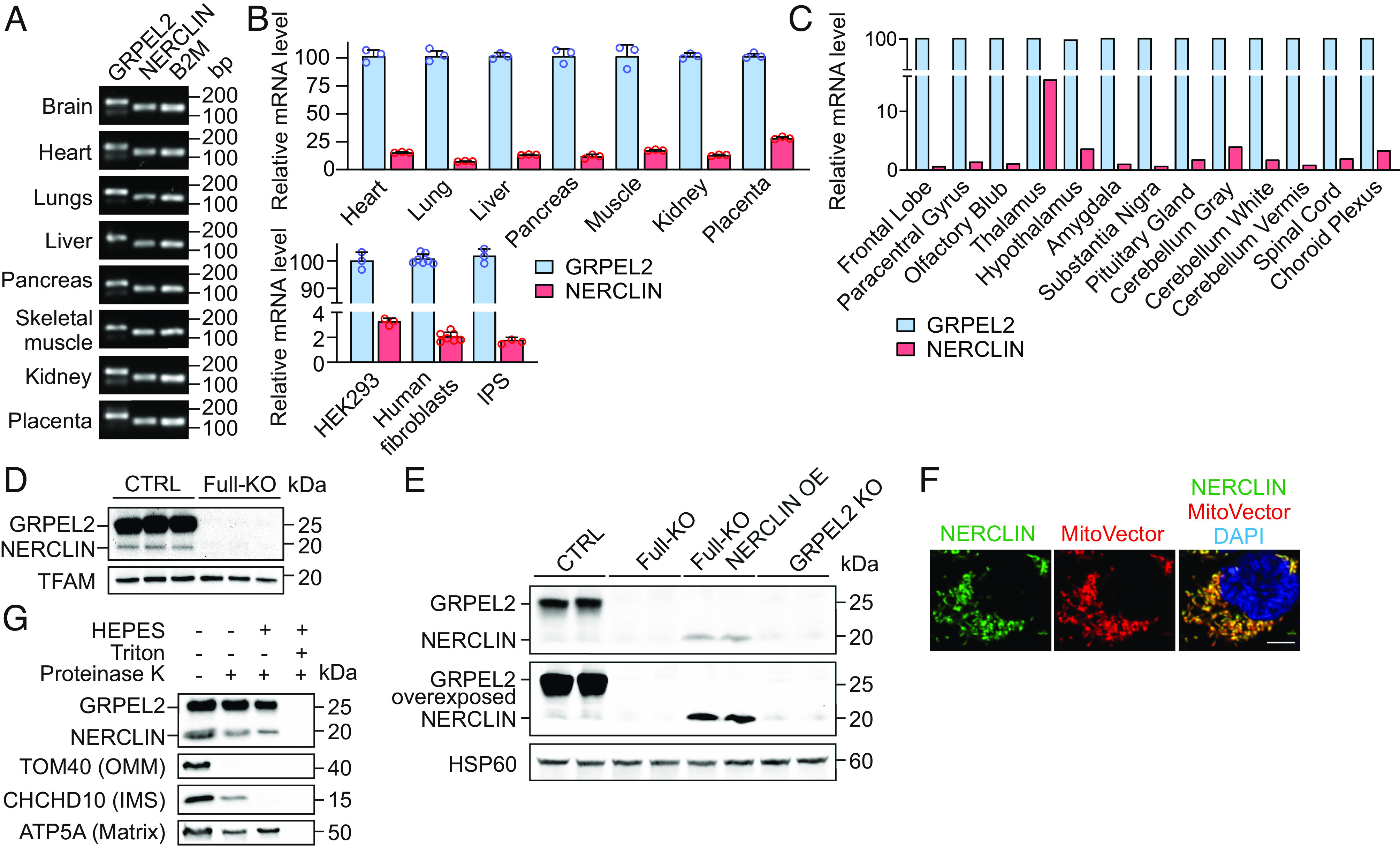
*NERCLIN* is expressed in human cells. (*A*) Expression pattern of *NERCLIN* mRNA in human tissues. Transcript-specific primer pairs indicated in Fig. 1*A* were used for PCR reaction. Gel electrophoresis of real-time PCR products. β2 microglobulin (B2M) was used as a loading control. (*B*) Relative mRNA levels of *NERCLIN* in human tissues and cultured cells as determined by qPCR. The mRNA level of *GRPEL2* was taken as 100%. n = 3 [human tissues, HEK293 cells, or human induced pluripotent stem cells (iPS)], n = 5 (human fibroblasts). (*C*) Relative mRNA levels of *NERCLIN* in human brain samples as determined by qPCR. The mRNA level of *GRPEL2* was taken as 100%. n = 1. (*D* and *E*) Protein expression of NERCLIN in mitochondria isolated from HEK293 cells. Full-KO cells lacking GRPEL2 and NERCLIN were used as negative control. TFAM or HSP60 was used as a loading control. Full-KO cells transfected with NERCLIN plasmid for 24 h (Full-KO NERCLIN OE) were used as positive control. (*F*) Intracellular localization of NERCLIN in 143B cells as shown by fluorescent microscopy. NERCLIN-EGFP construct and MitoVector Red were transiently coexpressed for 24 h. MitoVector Red was used for labeling mitochondria; DAPI shows nuclei. (Scale bar, 5 μm.) (*G*) Mitochondria isolated from HEK293 cells overexpressing NERCLIN were treated with proteinase K at isoosmotic (without HEPES) or hypoosmotic (with HEPES) conditions. Proteins were analyzed by western blotting. OMM, outer mitochondrial membrane, IMS, intermembrane space.

Quantitative RT-PCR analysis using primers specific for *GRPEL2* or *NERCLIN* showed that *NERCLIN* was expressed in all tested human tissues (Fig. 2 *A* and *B*), 13 different human brain regions (Fig. 2*C*), and in cultured human cells (Fig. 2*B*), suggesting that it is ubiquitously expressed. We estimate that *NERCLIN* expression is between 1% and 20% of *GRPEL2* expression level, depending on the tissue type or cell line (Fig. 2 *B* and *C*). We observed the highest relative expression level of approximately 20% in the placenta. Accordingly, data from the Genotype-Tissue Expression (GTEx) portal showed that *NERCLIN* is a low-abundance isoform of the *GRPEL2* gene in human tissues (*SI Appendix*, Fig. S1*C*).

The *GRPEL2* gene is found in vertebrates; however, EST databases contained transcripts lacking exon 3 only in some primates. We performed in silico analysis with genomic sequences to identify the nonhuman primates that had the potential for a conserved NERCLIN C terminus following exclusion of exon 3. We found sequences for a highly conserved C terminus in all apes and in some Old World monkeys and a less conserved C terminus in some New World monkeys, tarsiers, and strepsirrhines (*SI Appendix*, Fig. S1 *D* and *E*). As the green monkey was among the positive Old World monkeys, we performed RT-PCR analysis of green monkey kidney COS7 cells and indeed detected the *NERCLIN* transcript lacking exon 3 (*SI Appendix*, Fig. S1*F*). On the contrary, in RNA samples from the dog or mouse, only the product corresponding to *GRPEL2* could be amplified. These results suggest that NERCLIN is specific to a subgroup of primates.

Next, we investigated whether NERCLIN is effectively translated into a stable protein in vivo. We performed western blotting analysis of mitochondria isolated from HEK293 cells using an antibody directed against the GRPEL2 N terminus (residues 3-115). In addition to the 25 kDa GRPEL2 band, we detected a 15-kDa band putatively corresponding to NERCLIN (Fig. 2*D*). Importantly, both bands were absent in a full knockout (Full-KO) cell line lacking *GRPEL2* and *NERCLIN*, which we had previously generated by deleting exon 1 using CRISPR/Cas9 (21). To confirm that the second band was not a result of GRPEL2 protein degradation, we generated a *GRPEL2*-specific knockout cell line (GRPEL2 KO) in HEK293 cells by deleting the exon 3 using CRISPR/Cas9 (*SI Appendix*, Fig. S2 *A* and *B*). In GRPEL2 KO cells, the GRPEL2 band was absent, while the 15-kDa band stayed intact (Fig. 2*E*). As *NERCLIN* does not have any unique sequence, it is not possible to generate a cell line lacking *NERCLIN* without affecting *GRPEL2*. However, transient overexpression of NERCLIN in Full-KO cells confirmed that the 15-kDa band in western blot was NERCLIN (Fig. 2*E*). In line with the transcript levels, the endogenous NERCLIN protein was less abundant than GRPEL2.

NERCLIN shares the N-terminal MTS with GRPEL2, suggesting that NERCLIN is also a mitochondrial protein. Using fluorescent microscopy, we showed that NERCLIN-GFP fusion protein localizes strictly to mitochondria in human cells (Fig. 2*F*). To determine the submitochondrial localization of NERCLIN, we used the protease protection assay. NERCLIN was protected from protease after selective opening of the outer and inner mitochondrial membrane, suggesting that NERCLIN localizes to the mitochondrial matrix (Fig. 2*G*).

Altogether, these results show that NERCLIN is a small mitochondrial protein, which is expressed ubiquitously from the *GRPEL2* locus, but on a relatively low level, and found specifically in humans and some nonhuman primates.

### BioID Analysis of Proximal Proteins Suggests Distinct Functions for GRPEL2 and NERCLIN.

**Fig. 3.**
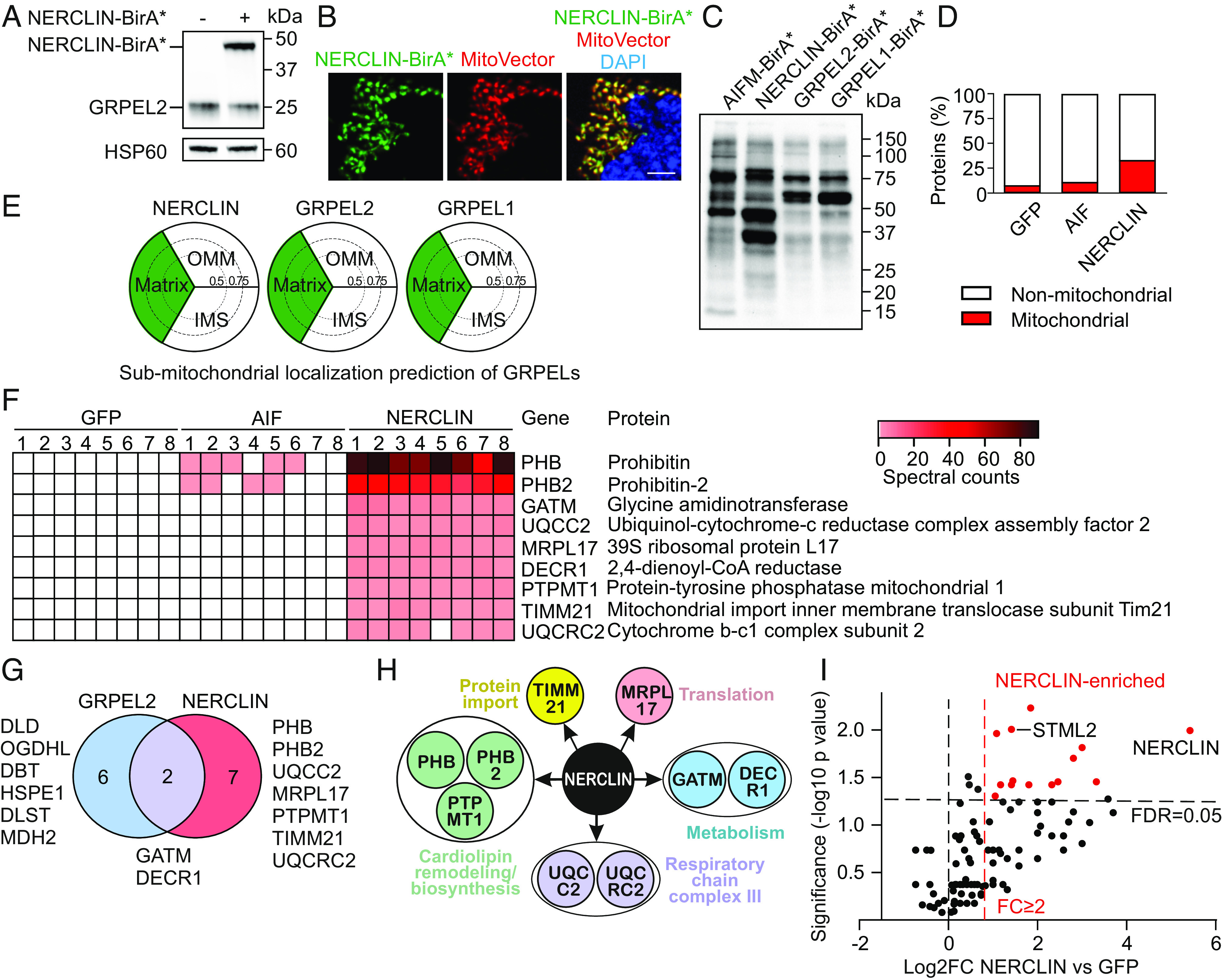
Proteins interacting with NERCLIN identified by BioID or IP. (*A*) Western blotting analysis of NERCLIN-BirA* expression in 143B cells. HSP60 was used as a loading control. 143B cells were transiently transfected with indicated constructs for 24 h. (*B*) Intracellular localization of NERCLIN-BirA* determined by immunocytochemistry. MitoVector Red was used to label mitochondria; DAPI shows nuclei. (Scale bar, 10 μm.) (*C*) Biotinylated proteins in total cell lysates analyzed by immunoblotting using anti-streptavidin antibody. 143B cells were transiently transfected for 24 h with indicated constructs. Biotin was added to the culture media for 24 h. (*D*) Percentage of the mitochondrial location of all significant proximal proteins of NERCLIN before filtering. (*E*) Interaction profile identified by BioID analysis was used for MS microscopy to predict suborganelle localization of NERCLIN. Three sectors in each plot represent the matrix, intermembrane space (IMS), and outer mitochondrial membrane (OMM). Sector areas indicate the possible location score of the proteins, with scores between 0 and 0.5, between 0.5 and 0.75, or 0.75 and 1 (see ref. 24) for the reference). (*F*) Heat map of the unique proximal proteins of NERCLIN (n = 8). (*G*) Venn diagram showing distinct unique proximal proteins for GRPEL2 and NERCLIN. (*H*) Classification of the proteins potentially interacting with NERCLIN identified by BioID. (*I*) Volcano plot of proteins identified by immunoprecipitation followed by mass spectrometry. 143B cells were transiently transfected with NERCLIN-Strep-HA or GFP-Strep-HA. A one-sided *t* test was performed for pairwise comparison. Proteins with a fold change ≥ 2 and a *P* value ≤ 0.05 (n = 3) are considered significantly enriched proteins and marked as red dots.

To gain insight into the function of NERCLIN in human cells, we analyzed its proximal proteins by the proximity-dependent biotin identification (BioID) approach (23). Promiscuous biotin ligase BirA* was fused to the C terminus of NERCLIN, and the expression of the fusion construct in human 143B cells was confirmed by immunoblotting (Fig. 3*A*). Immunocytochemistry showed that NERCLIN-BirA* fusion protein was localized to mitochondria (Fig. 3*B*). To exclude nonspecifically labeled proteins, we used BirA* fused to green fluorescent protein (GFP-BirA*) or to apoptosis-inducing factor (AIF-BirA*), a mitochondrial intermembrane space protein, as controls. Immunoblotting analysis following biotin labeling revealed that while the pattern of biotinylated proximal proteins for GRPEL1-BirA* and GRPEL2-BirA* was identical, confirming our previous findings (21), the pattern for NERCLIN-BirA* was different (Fig. 3*C*).

Biotinylated proteins from four replicates for each BirA* fusion protein were extracted using streptavidin beads and analyzed by mass spectrometry (Dataset S1). Two or more spectral matches in at least three replicates were considered significant hits and included in further analysis. NERCLIN-BirA* showed enrichment in proximal proteins localized to mitochondria (33%) compared to GFP-BirA* (10%) or AIF-BirA* (12%) (Fig. 3*D*). Using the BioID data and a previously described algorithm for mapping of protein localization (24, 25), we confirmed that similarly to GRPEL1 and GRPEL2, NERCLIN localizes to the mitochondrial matrix (Fig. 3*E*).

To identify highly specific proximal proteins of NERCLIN, we first excluded proteins detected in GFP or AIF control samples. Next, we excluded the mitochondrial matrix BioID matches that are commonly found with any matrix bait (21, 24). The resulting list of unique highly specific hits consisted of nine proteins that potentially interact with NERCLIN (Fig. 3*F* and Dataset S1). Notably, peptides unique to NERCLIN were specifically detected in NERCLIN-BirA* samples confirming NERCLIN expression (*SI Appendix*, Fig. S3). NERCLIN did not show proximity to GRPEL2 since no peptides unique to GRPEL2 were identified in NERCLIN-BirA* samples (*SI Appendix*, Fig. S3). This suggests that GRPEL2 and NERCLIN do not cross interact, although GRPEL2 is known to form homodimers (21).

Next, we compared the proximal proteins of GRPEL2 and NERCLIN. We used BioID data for GRPEL2 from our previous study where the same filtering was applied (21). Only two proteins were identified as shared potential interactors for GRPEL2 and NERCLIN, whereas six proteins were specific only to GRPEL2 and seven to NERCLIN (Fig. 3*G*). These findings suggested that GRPEL2 and NERCLIN function in distinct pathways in the human mitochondrial matrix.

Pathway analysis of the potential proteins interacting with NERCLIN revealed that three out of nine proteins were associated with CL synthesis or organization including both prohibitins (PHB and PHB2) and the mitochondrial protein-tyrosine phosphatase (PTPMT1) (Fig. 3 *F* and *H*). NERCLIN also showed proximity to some respiratory chain complex III and mitochondrial protein import and translation proteins.

As PTPMT1 was identified in proximity to NERCLIN by BioID analysis, we tested whether NERCLIN directly interacts with PTPMT1 using coimmunoprecipitation analysis. We did not observe coimmunoprecipitation of PTPMT1 with NERCLIN or vice versa (*SI Appendix*, Fig. S4*A*), suggesting that although in close proximity, NERCLIN does not directly interact with PTPMT1.

To reveal proteins with which NERCLIN forms stable complexes, we performed immunoprecipitation followed by mass spectrometry. For this experiment, 143B cells were transiently transfected with NERCLIN-Strep-HA or GFP-Strep-HA. Immunoprecipitation was performed on whole cell lysates from three replicates using anti-HA agarose beads, and the pull-down samples were analyzed by mass spectrometry. Nineteen proteins were specifically enriched in NERCLIN pull-down samples, among them was STML2, which interacts with prohibitins and the CL synthesis complex (17) (Fig. 3*I* and Dataset S2). By using immunoblotting, we confirmed that NERCLIN directly interacts with STML2 (*SI Appendix*, Fig. S4*B*). Thus, our unbiased BioID and immunoprecipitation analysis suggests that NERCLIN interacts with the CL synthesis complex.

### Overexpression of NERCLIN Disrupts Mitochondrial Morphology.

**Fig. 4.**
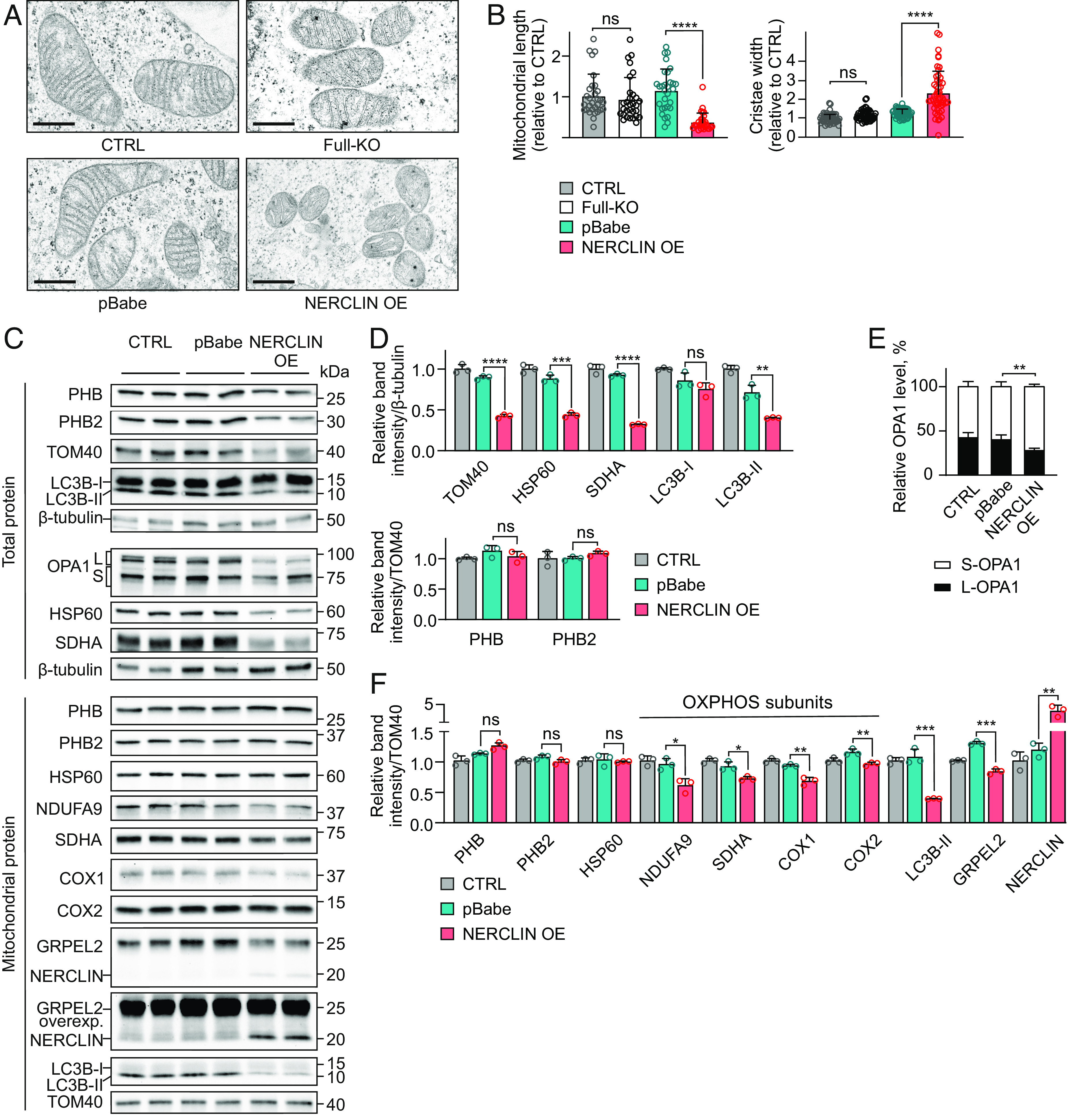
Overexpression of NERCLIN disrupts mitochondrial morphology and ultrastructure. HEK293 cells were transiently transfected with NERCLIN plasmid (NERCLIN OE) or with an empty vector (pBabe) for 48 h. CTRL, nontransfected cells. (*A*) Mitochondrial ultrastructure in HEK293 cells analyzed by transmission electron microscopy. Full-KO, cells lacking GRPEL2 and NERCLIN. (Scale bar, 500 nm.) (*B*) Quantitative analyses of mitochondrial major axis length (mitochondrial length, n = 30) and cristae width (n = 50) from electron microscopy images. (*C*) Western blot analysis of cells overexpressing NERCLIN. Total protein lysates or mitochondrial protein lysates were used. L, long OPA1 isoform, S, short OPA1 isoform. (*D*) Quantification of western blot images presented in (*C*). Protein expression levels are normalized to the β-tubulin level (n = 3). (*E*) Relative levels of short and long OPA1 isoforms determined by western blot analysis in (*C*). The total OPA1 level was taken as 100%. L, long OPA1 isoform, S, short OPA1 isoform (n = 4 for pBabe and NERCLIN OE; n = 7 for CTRL). (*F*) Quantification of western blot images presented in (*C*). Protein expression levels are normalized to the TOM40 level (n = 3). Data are shown as mean ± SD. *P < 0.05, **P < 0.01, ***P < 0.001, ****P < 0.0001, and ns, not significant (one-way ANOVA in B; unpaired *t* tests in *D*–*F*).

BioID data showed high spectral counts for the proximity of NERCLIN to PHB and PHB2. These two prohibitins assemble into heterodimers that form large ring-like complexes at the inner mitochondrial membrane, providing a scaffold for proteins and lipids that control cristae morphogenesis and functional integrity of mitochondria (26–28). We hypothesized that NERCLIN may affect mitochondrial morphology by its association with the prohibitins. We thus used electron microscopy on Full-KO cells and cells overexpressing NERCLIN. Interestingly, we observed that while cells without NERCLIN had a comparable mitochondrial morphology to nontransfected cells or cells transfected with empty vector (pBabe), the cells overexpressing NERCLIN had a severe disruption of mitochondrial ultrastructure (Fig. 4*A*). Particularly in NERCLIN overexpressing cells, the mitochondria were small and fragmented and had disordered cristae and irregular intermembrane and intercristal space (Fig. 4 *A* and *B*). Depletion of prohibitins is known to cause such a phenotype in cultured cells (29, 30). Next, we tested by immunoblotting if PHB or PHB2 levels were affected in cells overexpressing NERCLIN. The levels of all tested mitochondrial proteins were somewhat reduced after 48 h of overexpression, indicating that mitochondrial mass was decreasing in NERCLIN-expressing cells (Fig. 4 *C* and *D*). Nevertheless, the immunoblotting results did not indicate that NERCLIN would primarily affect prohibitins, as we did not detect a decrease in prohibitin levels relative to the mitochondrial marker TOM40 (Fig. 4 *C* and *D*) or at equal loading of proteins from isolated mitochondria (Fig. 4 *C* and *F*). Consistent with the morphological changes induced by NERCLIN, the immunoblotting analysis showed that NERCLIN overexpression reduced the level of long OPA1 (Fig. 4 *C* and *E*). In addition, we observed decreased levels of OXPHOS subunits in mitochondria isolated from cells overexpressing NERCLIN (Fig. 4 *C* and *F*). Autophagy marker LC3B-II was reduced by NERCLIN overexpression. (Fig. 4 *C* and *D*). Using live microscopy, we confirmed that NERCLIN overexpression causes mitochondrial fragmentation (*SI Appendix*, Fig. S5 *A* and *B*). NERCLIN overexpression also fragmented mitochondria in green monkey COS7 cells but not in mouse embryonic fibroblasts as determined by OPA1 western blot (*SI Appendix*, Fig. S6 *A–E*). These data indicate that NERCLIN regulates mitochondrial morphology specifically in primate cells.

Full-KO cells with or without stable overexpression of GRPEL2 did not show changes in mitochondrial mass, in the levels of OXPHOS subunits, or in the respiration rate (*SI Appendix*, Fig. S2 *C–J*), indicating that in contrast to NERCLIN overexpression, which causes severe disruption of mitochondrial ultrastructure, the lack of NERCLIN does not cause mitochondrial abnormalities under normal culture conditions.

### Overexpression of NERCLIN Reduces CL Levels.

**Fig. 5.**
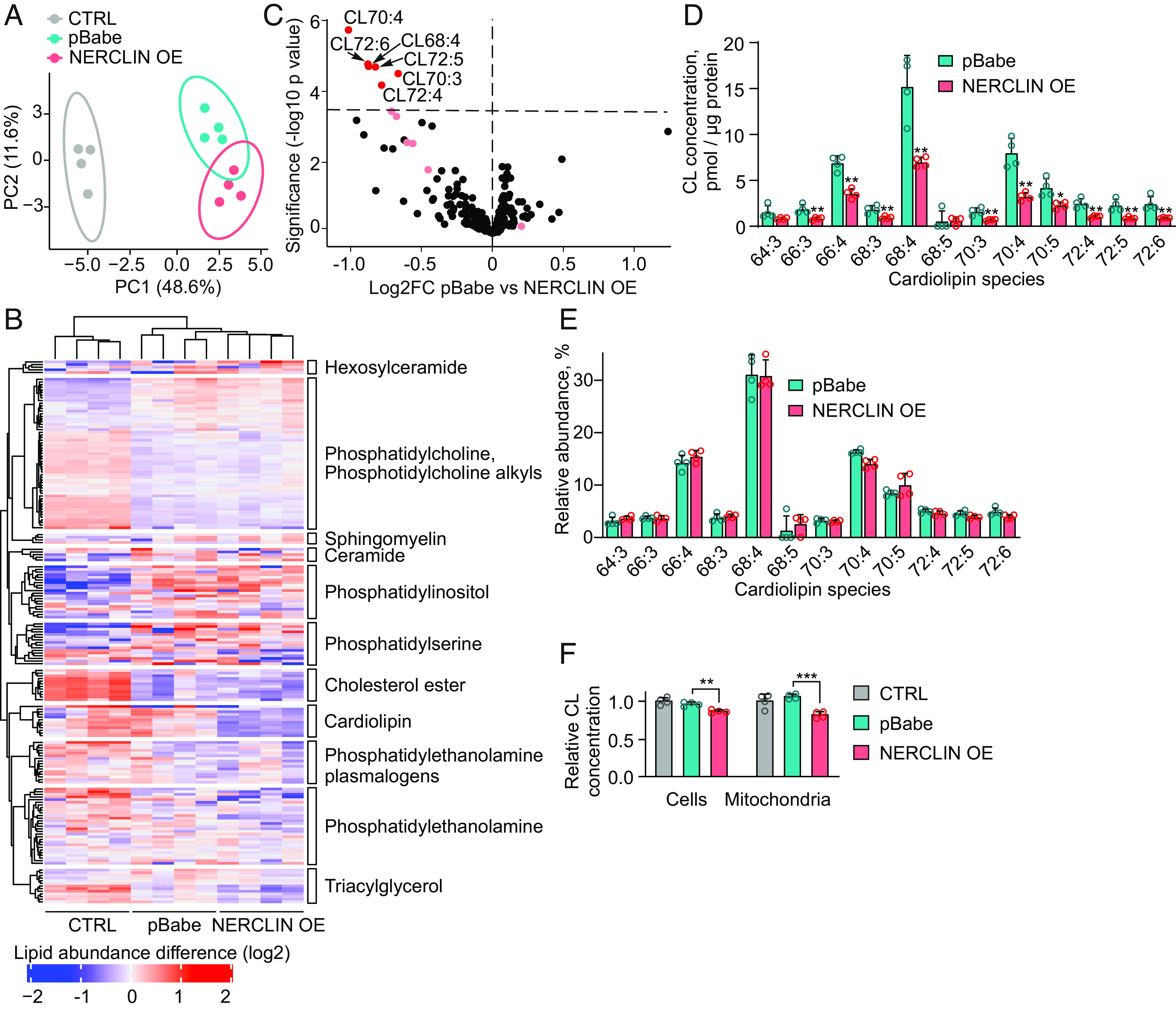
Overexpression of NERCLIN reduces CL levels. HEK293 cells were transiently transfected with NERCLIN plasmid (NERCLIN OE) or with an empty vector (pBabe) for 48 h. Lipids from four biological replicates for each sample were extracted and analyzed by mass spectrometry. CTRL, nontransfected cells. (*A*) Principal component analysis (PCA) in a two-dimensional score plot of control (CTRL), NERCLIN overexpressing cells, and cells transfected with empty vector (pBabe). The first two principal components are shown on the *X* axis and *Y* axis, explaining 48.6% and 11.6% of variance, respectively. The circles represent 95% confidence regions. (*B*) Heat map showing the relative lipid concentrations of individual species in control (CTRL), NERCLIN overexpressing cells, and cells transfected with empty vector (pBabe). Each column represents one sample, and each line represents one compound colored by its normalized abundance. Lipid abundance ratios are colored according to the fold changes, and the color key indicates the magnitude of log2 fold change. (*C*) Volcano plot comparing the lipid composition of NERCLIN overexpressing cells and cells transfected with empty vector (pBabe). The dashed line indicates a false discovery rate (FDR)-corrected *P* value of 0.05. Significantly changed lipids are in red and others in black. Light red indicates CL species that are not significantly changed. (*D*) CL concentrations in cells transfected with NERCLIN or empty vector (pBabe). (*E*) CL profile of NERCLIN overexpressing cells compared to cells transfected with empty vector (pBabe). The abundance of individual CL species is normalized to total CL content. (*F*) Total CL concentration in HEK293 cells or in mitochondria isolated from HEK293 cells transfected with NERCLIN or empty vector (pBabe) as determined by fluorometric assay (n = 4). In all graphs, data are presented as mean ± SD. **P < 0.01, ***P < 0.001, and ns, not significant as compared to the cells transfected with empty vector (unpaired *t* tests).

Our results suggesting that NERCLIN interacts with the CL synthesis complex prompted us to investigate whether CL maintenance was affected by NERCLIN overexpression. Principal component analysis of lipid profiles from lipidomics analysis showed that transfection (empty vector or NERCLIN) had a major effect on cellular lipids. However, cells overexpressing NERCLIN also separated from the cells transfected with empty vector (pBabe) based on their lipid profile (Fig. 5*A*). Interestingly, the analysis of 189 individual lipid species from 11 lipid classes showed a significant reduction of six CL species in cells overexpressing NERCLIN compared to the cells transfected with empty vector (Fig. 5 *B* and *C* and Dataset S3), while none of the other lipid species were significantly altered. Analysis of the CL profile suggested no accumulation or depletion of a specific CL species upon overexpression of NERCLIN (Fig. 5 *D* and *E*) but a general reduction in CL content. This observation suggested that the overall CL synthesis was affected by NERCLIN and not a particular step in CL maturation. Since CL is located almost exclusively in the mitochondrial inner membrane, the reduced cellular CL level could reflect the decrease of mitochondrial content, which we had observed by western blotting of mitochondrial proteins in cells overexpressing NERCLIN (Fig. 4 *C* and *D*). To exclude that the reduction in CL level was solely an indication of the decreased mitochondrial mass, we measured total CL in HEK293 cells and in isolated mitochondria using a fluorometric assay. We found the CL level to be reduced both in total cells and in isolated mitochondria as a result of NERCLIN overexpression (Fig. 5*F*), indicating that NERCLIN specifically depleted mitochondrial CL. In line with the unaltered mitochondrial morphology in the absence of NERCLIN, the levels of CL species were normal in Full-KO cells (*SI Appendix*, Fig. S2*E*).

### NERCLIN Is Responsive to Heat Stress.

**Fig. 6.**
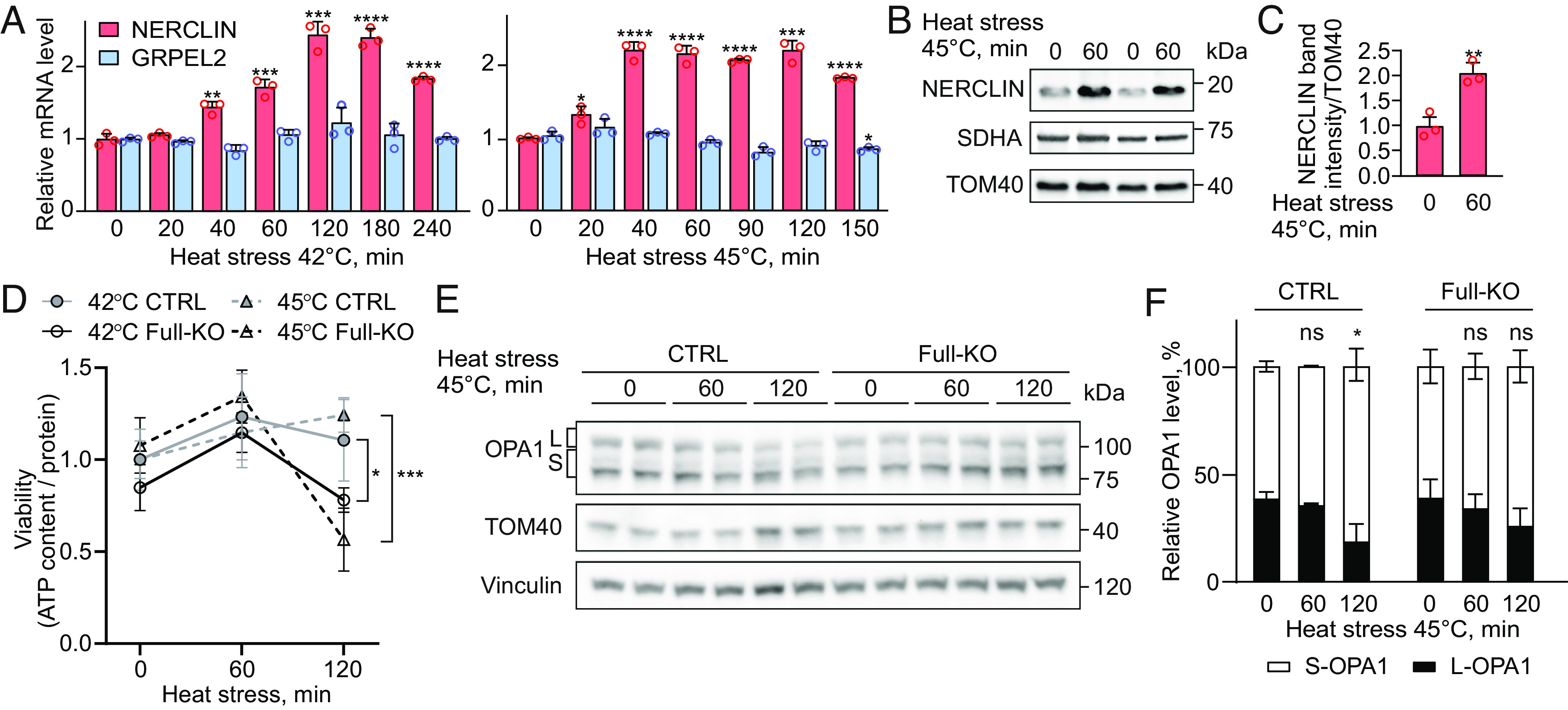
NERCLIN protects cells from heat stress. (*A*) mRNA levels of *NERCLIN* or *GRPEL2* in HEK293 cells exposed to heat stress (42 °C or 45 °C) as determined by the qPCR assay (n = 3). (*B*) NERCLIN protein levels in GREPL2 KO HEK293 cell exposed to heat stress (45 °C) were analyzed by western blot. Mitochondrial protein extracts were used for the analysis. TOM40 was used as a loading control. (*C*) Quantification of western blot images presented in (*B*). The NERCLIN protein level is normalized to TOM40 level (n = 3). (*D*) Viability of HEK293 control cells (CTRL) or cells lacking GRPEL2 and NERCLIN (Full-KO) exposed to heat stress (42 °C or 45 °C). The ATPlite viability assay kit was used to analyze cell viability. ATP content was normalized to protein levels. The viability relative to the untreated CTRL cells is shown. (*E*) Western blot analysis of HEK293 control cells (CTRL) or cells lacking GRPEL2 and NERCLIN (Full-KO) exposed to heat stress (45 °C). L, long OPA1 isoform, S, short OPA1 isoform. TOM40 and vinculin were used as loading controls. (*F*) Relative levels of short and long OPA1 isoforms determined by western blot analysis in (*E*). The total OPA1 level was taken as 100%. L, long OPA1 isoform, S, short OPA1 isoform (n = 3). Data are presented as mean ± SD. *P < 0.05, **P < 0.01, ***P < 0.001, ****P < 0.0001, and ns, not significant as compared to control, untreated cells (unpaired *t* tests).

Using cells lacking both GRPEL2 and NERCLIN, we showed that NERCLIN is not an essential protein in human cells under normal culture conditions (Fig. 2*D*). To investigate whether NERCLIN is responsive in stress conditions, we subjected HEK293 cells to heat or oxidative stress. We observed a significant induction of *NERCLIN* mRNA expression (up to 2.3-fold change) in heat stress (Fig. 6*A*) but not in oxidative stress (*SI Appendix*, Fig. S7*A*). Importantly, *GRPEL2* mRNA expression was unchanged in heat stress. To analyze the NERCLIN protein level in response to heat stress, we used HEK293 cells lacking GRPEL2 (GRPEL2 KO) to exclude the possibility to detect degraded GRPEL2 protein near the NERCLIN protein band by western blotting. We showed that heat stress induced the NERCLIN protein level (up to twofold change) (Fig. 6 *B* and *C*).

To investigate the biological significance of NERCLIN induction under heat stress, we exposed control or Full-KO HEK293 cells to milder (42 °C) or severe (45 °C) heat stress. We showed that Full-KO cells were more sensitive to heat stress as determined by live microscopy (*SI Appendix*, Fig. S7*B*) and viability assay (Fig. 6*D*). To demonstrate that the lack of NERCLIN was responsible for elevated heat-induced cell death in Full-KO cells, we performed heat stress experiments using GRPEL2 KO cells. In contrast to Full-KO cells, GRPEL2 KO cells were heat resistant (*SI Appendix*, Fig. S7*C*), suggesting that NERCLIN specifically protects cells against heat stress.

To further test the protective role of NERCLIN under heat stress, we performed a heat experiment using control cells overexpressing NERCLIN. We showed that cells overexpressing NERCLIN are more resistant to heat stress (42 °C, 2 h) compared to the cells transfected with empty vector (*SI Appendix*, Fig. S7*D*).

As we showed that NERCLIN overexpression reduced the level of long OPA1, we analyzed OPA1 isoforms in Full-KO HEK293 cells exposed to heat stress. In agreement with the previous studies (31, 32), heat stress triggered the processing of long OPA1 in HEK293 cells (Fig. 6 *E* and *F*). However, Full-KO cells did not show significant changes in the levels of OPA1 isoforms under heat stress (Fig. 6 *E* and *F*). In addition, we showed that heat stress induced the lipidation of LC3B in both control and Full-KO cells (*SI Appendix*, Fig. S7 *E–H*). However, in contrast to the control cells, the LC3B-I level in Full-KO cells was not reduced by heat stress.

To test whether mitophagy contributed to heat-induced cell death, we inhibited mitophagy prior to heat stress using chloroquine. The inhibition of mitophagy did not sensitize control or Full-KO cells to heat stress (*SI Appendix*, Fig. S8 *A*–*E*), suggesting that although mitophagy is responsive to heat, it does not contribute to cell death under heat stress.

To investigate whether NERCLIN contributes to heat stress adaptation by regulating CL, we performed lipidomics analysis on control or Full-KO cells exposed to heat stress. We observed that under heat stress, cells lacking NERCLIN tended to have higher CL levels compared to the control cells; however, the difference was not statistically significant (*P* = 0.0816) (*SI Appendix*, Fig. S7*I* and Dataset S4). In conclusion, *NERCLIN* expression is induced by heat, and cells lacking NERCLIN are more sensitive to heat stress. NERCLIN is required for heat stress–induced OPA1 processing, suggesting that NERCLIN protects against heat stress by regulating mitochondrial dynamics.

## Discussion

We have here identified and characterized a human mitochondrial matrix protein, NERCLIN. It represents a small protein, being 90 amino acids in its mature form after MTS cleavage. NERCLIN is transcribed from the *GRPEL2* locus; however, it does not have the properties of a GrpE-like nucleotide exchange factor. Instead, NERCLIN is likely to be a small alpha-helical protein, which we find to locate in the proximity of the prohibitins and the CL synthesis complex at the matrix side of the inner mitochondrial membrane. NERCLIN is ubiquitously expressed in human cells and tissues, and its normally relatively low expression level appears to be tightly regulated. The absence of NERCLIN does not cause any obvious defects in the mitochondria of cultured cells under normal culture conditions, but its overexpression leads to dramatic changes in mitochondrial structure and cristae maintenance mediated by long OPA1 processing. These changes associated with a specific reduction in CL levels. Thus, we propose NERCLIN as a negative regulator of CL and mitochondrial ultrastructure.

Mitochondrial proteome is enriched in small proteins, which was speculated to be energetically favorable considering the need to import the proteins through the double membranes (9). Another reason may be the wide requirement for regulation of mitochondrial form and function depending on a tissue and cell state, for which a range of small proteins may have evolved to contribute.

We demonstrated that NERCLIN is responsive to heat stress ensuring OPA1 processing and cell survival. Mitochondrial fission and fragmentation caused by OPA1 degradation have an adaptative and protective role in stress response maintaining bioenergetics homeostasis (33, 34). We propose that heat-induced elevation of NERCLIN induces the adaptive OPA1 processing and mitochondrial fragmentation thus protecting from cell death. Consistent with our results, the heat stress resistance in worms is increased by induced mitochondrial fragmentation (35). Furthermore, enhanced mitochondrial fragmentation was shown to promote the viability of cancer cells under stress conditions (36–38).

Our unbiased BioID, immunoprecipitation, and lipidomics results point to NERCLIN having interactions in the site where prohibitin complexes form scaffolds for CL synthesis (39). STML2, identified in our study as a direct interactor of NERCLIN, is known to regulate mitochondrial biogenesis and function by recruiting prohibitins to cardiolipin (17). Thus, NERCLIN potentially regulates CL levels and mitochondrial morphology through its interaction with STML2. STML2 is required for stress-induced mitochondrial hyperfusion, an adaptive prosurvival response triggered by some types of stress such as apoptotic stimuli (40). In contrast, heat stress response requires mitochondrial fragmentation (31, 32), suggesting that mitochondrial morphology adjusts specifically to a certain mode of stress. We propose that under heat, NERCLIN inhibits STML2, promoting mitochondrial fission and cell survival. Indeed, we showed that NERCLIN is required for heat stress–induced OPA1 processing, and cells lacking NERCLIN tended to have higher CL levels under heat stress. Thus, NERCLIN potentially fine-tunes the mitochondrial stress response. Further studies are required to identify the precise mechanisms of NERCLIN-mediated mitochondrial fragmentation.

Interestingly, we showed rapid and significant induction of the *NERCLIN* mRNA level already after 40 min of heat treatment, whereas the *GRPEL2* mRNA level was unchanged. Considering that both transcripts are encoded by the same gene, these results suggest that the *NERCLIN* induction under heat stress is regulated on the mRNA splicing level rather than the transcriptional level.

Another recently identified small mitochondrial protein, mitoregulin, is also linked to CL (11). In contrast to NERCLIN, mitoregulin has positive effects on mitochondrial functions, such as on respiratory supercomplex assembly, and it is found widely in vertebrates. The loss of PTPMT1, one of the essential components of the CL synthesis complex that we found in proximity of NERCLIN, results in mitochondrial fragmentation and abnormal mitochondrial morphology (41) similar to the phenotype that we observed in cells overexpressing NERCLIN. CL has a profound importance for mitochondrial function and dynamics, requiring a complex regulation, and thus, NERCLIN may have evolved to adjust CL biosynthesis to precise cellular needs. Interestingly, *TAZ* encoding mitochondrial acyltransferase tafazzin, which mediates CL maturation, also has a primate-specific splice variant (42, 43). In *Arabidopsis*, CL was shown to mediate mitochondrial dynamics and heat stress response (44, 45). Thus, the heat-induced elevation of NERCLIN may inhibit CL biosynthesis and cause mitochondrial fragmentation (33, 34).

In conclusion, NERCLIN identified in this study significantly differs from GRPEL2 by its expression, structure, function, and regulation in stress conditions. Similar to many other small proteins, NERCLIN has been overlooked in the previous transcriptomic and proteomic studies due to its small size and partial overlap with the GRPEL2 gene and protein. Nevertheless, our study shows that these small proteins can have important regulatory functions.

## Materials and Methods

### Cell Culture.

This study involves human fibroblasts and iPS cells derived from healthy donors. Human stem cell research was approved by the Coordinating Ethics Committee of the Helsinki and Uusimaa Hospital District (Nr 95/13/03/00/15). All the cell lines were anonymized.

### DNA and RNA Analysis.

For expression analysis, human multiple tissue cDNA panel (MTC™ Clontech, 636742) and human normal brain tissue qPCR array (OriGene Technologies, HBRT101) were used. Prior to their use, these samples were deidentified by manufacturers.

Detailed methods are described in *SI Appendix*, *Materials and Methods*.

## Supplementary Material

Appendix 01 (PDF)

Dataset S01 (XLSX)

Dataset S02 (XLSX)

Dataset S03 (XLSX)

Dataset S04 (XLSX)

Dataset S05 (XLSX)

Dataset S06 (XLSX)

## Data Availability

The main data supporting the findings of this study are available within the article and as *SI Appendix*. Unprocessed western blot images and numerical data are available in *SI Appendix* (Dataset S6). The raw proteomics data obtained in this study are available in the MassIVE under the ID MSV000086786 (46).
