# Supplementary material for: Small mitochondrial protein NERCLIN regulates cardiolipin homeostasis and mitochondrial ultrastructure
Source: Proc Natl Acad Sci U S A. 2023 Jul 18;120(30):e2210599120. doi: 10.1073/pnas.2210599120 (PMC10372682; doi:10.1073/pnas.2210599120)
Supplement: Supplementary file 1 — Appendix 01 (PDF) [file pnas.2210599120.sapp.pdf]

PNAS

# Supporting Information for

## Small mitochondrial protein NERCLIN regulates cardiolipin homeostasis and mitochondrial ultrastructure

Svetlana Konovalova, Rubén Torregrosa-Muñumer, Pooja Manjunath, Xiaonan Liu, Sundar Baral, Kaneez Fatima, Minna Holopainen, Jouni Kvist, Jayasimman Rajendran, Yang Yang, Markku Varjosalo, Reijo Käkelä, Pentti Somerharju, Henna Tyynismaa

Svetlana Konovalova
Email: svetlana.konovalova@helsinki.fi

**This PDF file includes:**

Materials and methods
Figures S1 to S8

**Other supporting materials for this manuscript include the following:**

Dataset S1 to S6

## Materials and methods

### Prediction of NERCLIN structure

NERCLIN protein secondary structure was predicted using Chou and Fasman Secondary Structure Prediction server, CFSSP (https://www.biogem.org/tool/chou-fasman), Jpred4 (http://www.compbio.dundee.ac.uk/jpred4) (1), secondary structure prediction method GOR4 (2), PSIPRED server (3) and PHD (https://npsa-prabi.ibcp.fr/cgi-bin/npsa_automat.pl?page=/NPSA/npsa_phd.html). The tertiary structure of human NERCLIN was predicted by SSpro server (http://scratch.proteomics.ics.uci.edu/). For the analysis mitochondrial targeting sequence (1-32 amino acids) was removed from the protein sequence.

### *In silico* analysis of *GRPEL2* sequences in primates

The mRNA sequences were extracted from UCSC Genome Browser (4). The sequences corresponding to exon 3 were removed and the obtained sequences lacking exon 3 were translated to amino acid sequence. The amino acid sequences of *GRPEL2* gene skipping exon 3 were aligned using ClustalW algorithm.

### DNA and RNA analysis

For expression analysis human multiple tissue cDNA panel (MTC™ Clontech, 636742) and human normal brain tissue qPCR array (OriGene Technologies, HBRT101) were used. Prior to their use these samples were de-identified by manufacturers. 1-3 ng of cDNA was used for real-time PCR assay. After completion of PCR amplification of the samples from a human multiple tissue cDNA panel, the PCR products were loaded on 0.7% agarose gel for electrophoresis. Isoform expression of GRPEL2 was analyzed using the data in the Genotype-Tissue Expression (GTEx) Project (https://gtexportal.org/home/).

### Real-time PCR assay

Total RNA was extracted from cultured cells using Mini spin kit (Macherey-Nagel). RNA extracted from canine fibroblasts was obtained from Prof. Hannes Lohi's group. 1000 ng of RNA was reverse transcribed using Maxima First Strand cDNA Synthesis Kit for RT-qPCR (Thermo Fisher Scientific, K1641). Real-time PCR analysis was done by DyNAmo Flash SYBR Green qPCR Kit (Thermo Fisher Scientific, F-415L) using CFX96™ Real-Time PCR Detection System (Bio-Rad). The PCR program started with 95°C for 7 min followed by 40 cycles at 95°C for 10 s and 60°C for 30 s. Beta-2-Microglobulin (B2M) was used as a reference gene for normalization and the mRNA expression level was calculated using the comparative Ct (threshold cycle) method. All primer sequences are in Dataset S5.

### Cell culture

Human embryonic kidney cells (HEK293), human osteosarcoma cells (143B), human fibroblasts, mouse embryonic fibroblasts (MEFs) or African green monkey kidney fibroblasts (COS7) were cultured (37°C, 5% CO2) in DMEM (Lonza, BE12-614F), supplemented with 10% fetal bovine serum (Life Technologies, 10270106), l-glutamine (Gibco, 25030024) and penicillin/streptomycin (Gibco, 15140122). Previously characterized(5) human induced pluripotent stem cells (iPS) were maintained on Matrigel-coated (Corning, 356231) plates with E8-medium supplemented with E8-supplement (Gibco, A1517001) and 50 µg/mL uridine (Sigma, U3003). iPS cells were passaged with 0.5 mM EDTA (Invitrogen, 15575020) in PBS (Life Technologies, BE17-516F) when 70% confluent. iPS cells at passage number 33 were used in the experiments. To induce heat stress, HEK293 cells were incubated at 42°C or 45°C for indicated times in a cell incubator in normoxia and 5% CO2. Control cells were kept at 37°C. After the treatment, representative images were taken using a bright field microscope, and the samples were processed immediately. To induce oxidative stress HEK293 cells were treated with 250 µM or 500 µM $H_2O_2$ (Sigma, H1009) for the indicated times. After the treatment, the cells were scraped, pelleted at 1000 g for 10 min and washed once with cold PBS (Life Technologies, BE17-516F). To inhibit mitophagy we used chloroquine (Sigma, C6628). JetPRIME reagent (Polyplus, 114-15) was used for cell transfection. After transfection cells were cultured for 24 or 48 h. This study involves human fibroblasts and iPS cells derived from healthy donors after providing written consent for the use of patient

material. Human patient stem cell research was approved by the Coordinating Ethics Committee of the Helsinki and Uusimaa Hospital District (Nr 95/13/03/00/15).

**Generation of stably expressing cell lines**

To generate stably overexpressing cell lines retroviral constructs were transiently transfected into phoenix package cell line with jetPRIME transfection reagent (Polyplus, 114-15) and HEK293 cells were infected by being exposed to virus-containing medium in the presence of polybrene (Millipore, TR-1003). Infected cells were treated with 1.5 µg/ml puromycin (Sigma, P8833) for five days until resistant clones formed. The expression of the constructs was confirmed by immunoblotting or/and by qPCR analysis.

**Mitochondria isolation**

Cultured HEK293 cells were scraped and harvested at 1000 g for 10 min at 4 °C. Cell pellets were washed with PBS (Life Technologies, BE17-516F), resuspended in ice-cold mitochondria isolation buffer (200 mM mannitol, 70 mM sucrose, 10 mM HEPES, 1mM EGTA, 0.2 % delipidated BSA (Biowest, P6154), protease inhibitor cocktail (Thermo Scientific, 78444), pH 7.5) and homogenized with 60 strokes using Dounce glass-glass homogenizer. Nuclei and unbroken cells were removed by centrifugation at 600 g for 20 min. The supernatant was centrifuged at 10 000 g for 10 min to pellet mitochondria. Mitochondrial pellets were resuspended in mitochondria isolation buffer without BSA and pelleted at 10 000 g for 10 min.

**Plasmid construction**

To clone the NERCLIN cDNA we extracted total RNA from 143B cells using Mini spin kit (Macherey-Nagel, 740955.50). Then RNA was reverse transcribed using Maxima First Strand cDNA Synthesis Kit for RT-qPCR (Thermo Scientific, K1641). The cDNA was PCR amplified using specific primers for TOPO cloning. Following gel electrophoresis, the correct band was cut, and the DNA was extracted from the gel for TOPO cloning. The TOPO construct containing NERCLIN was used as a template for cloning NERCLIN into EGFP or pBabe vectors. Briefly, NERCLIN without stop codon was amplified from TOPO vector containing NERCLIN and cloned into pEGFP-N1 vector using EcoRI/AgeI restriction sites. To generate NERCLIN-pBabe, full-length NERCLIN was amplified from TOPO vector containing NERCLIN and cloned into pBabe using EcoRI/SalI restriction sites.

To fuse BirA* to C-terminus of NERCLIN we used PCR overlap extension. pHA-BirA* and pBabe-NERCLIN constructs were used as templates. The resulting fused construct was inserted into pBabe vector using EcoRI and SalI restriction sites. GFP-BirA* and AIF-BirA* plasmids were as described in (6). The constructs for co-immunoprecipitation experiments (PTPMT1-V5, NERCLIN-Strep-HA, PTPMT1-Strep-HA, and NERCLIN-V5) were generated by the Genome Biology Unit supported by HiLIFE and the Faculty of Medicine, University of Helsinki, and Biocenter Finland. All plasmids were confirmed by Sanger sequencing. Primers used for the cloning are in Dataset S5.

AcGFP1-Mito (MitoGFP) and pDsRed2-Mito Vector (MitoVector Red) were purchased from Clontech (Mountain View, CA).

**Proteinase K accessibility assay**

Mitochondria were isolated as described in (7) and the proteinase K accessibility assay was performed as previously described (8). Briefly, after 24 h transient overexpression of NERCLIN cells from at least two 15-cm dishes were harvested. Cells were washed once with cold PBS (Life Technologies, BE17-516F) and spun at 500 g for 3 min at 4ºC. The cell pellet was resuspended in 10 mL of ice-cold extraction buffer (10 mM Tris-Mops, pH 7.4, 1 mM EGTA-Tris, pH 7.4, 0.2 M sucrose (Sigma, S0389), pH adjusted to 7.4) with 1x protease inhibitor cocktail (Thermo Scientific, 78444), and cells were disrupted using a glass-glass homogenizer. Unbroken cells were spun by centrifugation at 600 g for 10 min at 4ºC, and the pellet was subjected to the second round of homogenization. Mitochondria were recovered from the supernatant of both rounds of homogenization by centrifugation at 7 000 g for 10 min at 4ºC, and the pellet was washed once with a cold extraction buffer and centrifuged again. The final pellet was resuspended in 200 µL of extraction buffer and the mitochondrial protein concentration was

determined with bicinchoninic acid (BCA) Protein Assay Kit (Thermo Scientific, 23227). For the Proteinase K accessibility assay, 50 µg of mitochondria were resuspended in 100 µL of extraction buffer (untreated), extraction buffer and Proteinase K (protease-treated mitochondria), 2 mM HEPES (Life Technologies, 15630-049), pH 7.4 and Proteinase K (protease-treated mitoplasts), or 2 mM HEPES, pH 7.4, 0.1% Triton X-100 (Thermo Scientific, 11473613) and Proteinase K (protease- and detergent-treated mitoplasts). 100 µg/mL proteinase K (Thermo Scientific, EO0491) was used in all cases. Samples were incubated on ice for 30 min and the reaction was inactivated with a final concentration of 1mM PMSF (Sigma, 93482). Samples were precipitated with 10% trichloroacetic acid, washed once with cold acetone and resuspended in 50 µL of 4 × Laemmli sample buffer (BioRad, 1610747) containing 4% (vol/vol) β-mercaptoethanol (BioRad, 1610710) and protease inhibitor cocktail (Thermo Scientific, 78444). Subsequently, 10 µL of each sample were boiled at 95ºC for 5 min and separated by SDS-PAGE, followed by western blotting analysis (described in 'Western blotting').

**Immunocytochemistry**
143B cells were plated to the coverslips in 6-well plates. To label mitochondria, the cells were transiently transfected with pDsRed2-Mito Vector (Clontech, 632421). In the experiments for studying intracellular localization of NERCLIN, the cells were co-transfected with the NERCLIN-EGFP construct. In the experiments with BirA* fused proteins, the cells were co-transfected with NERCLIN-BirA*. After 24 h of transfection, the cells were fixed with 4% paraformaldehyde for 10 min at RT and washed with PBS (Life Technologies, BE17-516F). Then the cells were permeabilized with Triton X-100 (Thermo Scientific, 11473613) for 15 min at RT, washed, and blocked with 5 % BSA (Jackson ImmunoResearch, 001-000-162) for 2 h at RT. Cells were then incubated with corresponding primary antibody against GRPEL2 (Novus Biological, NBP1-85099, 1:50) in a blocking buffer overnight at +4 ºC. After washing cells were incubated with secondary antibody for 1 h at RT (Alexa Fluor 488 goat anti-rabbit, Invitrogen, R37116, 1:400). Finally, the cells were washed, mounted using antifade mounting medium containing DAPI (Vector Laboratories, H-1000) and imaged with Axio Observer Z1 (Zeiss).

**Generation of gene knockouts**
CRISPR/Cas9 was used to generate knockout HEK293 cells. Cells were co-transfected with two gRNA transcriptional cassettes prepared by PCR and CAG-Cas9-T2A-EGFP plasmid (Addgene, 7831) as described elsewhere (9). To generate a Full KO cell line lacking *GRPEL2* and *NERCLIN*, one of the guide RNAs was targeted to the 5' UTR region and the other to the beginning of exon 1. To generate *GRPEL2*-specific knockout cell line one of the guide RNAs was targeted to the intron 2 region and the other to intron 3 of the *GRPEL2* gene to remove exon 3. After 24 h of transfection GFP-positive cells were sorted by FACS and single-cell clones were generated. The sequences of gRNAs are in Dataset S5.

**Western blotting**
Whole cells or mitochondrial samples were lysed in RIPA buffer (Cell Signaling, 9806S) supplemented with protease inhibitor cocktail (Thermo Scientific, 78444). Following 10 min incubation on ice, the samples were centrifuged at 14 000 g for 10 minutes at +4°C. Protein concentration was measured by bicinchoninic acid (BCA) assay (Thermo Fisher, 23227). Protein lysates were supplemented with a Laemmli sample buffer (Bio-Rad, 1610747) containing 4% (vol/vol) β-mercaptoethanol (BioRad, 1610710) and resolved on 10 % Mini-PROTEAN TGX Precast Gels (Bio-Rad, 4561033/4561036). To detect OPA1 isoforms proteins were resolved on 7.5% Mini-PROTEAN TGX Precast Gels (Bio-Rad, 4561023). Then proteins were transferred to a 0.2-µM PVDF membrane (Bio-Rad, 1704156) by Trans-Blot Turbo Transfer System (Bio-Rad). The membranes were blocked in 5% milk in TBS–Tween 20 (0.1%) for 1 h at room temperature. Proteins were immunoblotted with the indicated primary antibodies in TBS–Tween 20 (0.1%) containing 1% BSA (Biowest, P6154) overnight, at 4 °C. Following primary antibodies were used: anti-GRPEL2, 1:1000 (Novus, 90536); anti-TFAM, 1:1000 (Abcam, 131607); anti-PHB, 1:1000 (Boster Bio, PA1932); anti-PHB2, 1:1000 (BioLegend, 611802); anti-beta-tubulin, 1:1000 (Cell Signaling, 2146); anti-NDUFA9 1:2000 (Abcam, 14713); anti-SDHA 1:2000 (Abcam, 14715); anti-COX1 1:2000 (Abcam, 14705); anti-HSP60 1:2000 (Santa Cruz, 1052); anti-LC3B 1:1000 (Novus

biologicals, 600-1384); anti-ATP5A 1:1000 (Abcam, 14748); CHCHD10 1:1000 (Sigma, HPA003440); anti-COX2 1: 1000 (GeneTex, GTX62145); anti-TOM40 1:1000 (Santa Cruz, 11414); anti-OPA1 1:1000 (BD Biosciences, 612606); anti-vinculin 1:10000 (Sigma, V9264); anti-HA tag 1:1000 (Cell Signaling, 3724); anti-V5 tag 1:1000 (Abcam, 9116); anti-STML2 1:1000 (Proteintech, 10348-1-AP). Membranes were extensively washed and probed with HRP conjugated secondary antibodies against mouse (Jackson ImmunoResearch, 111-035-146), rabbit (Jackson ImmunoResearch, 111-035-144) or goat IgG (Jackson ImmunoResearch, 305-035-003) at 1:5000 in TBS–Tween 20 (0.1%) containing 1% BSA (Biowest, P6154) and washed again. To detect biotinylated proteins, the membrane was incubated with anti-streptavidin-HRP antibody (Jackson ImmunoResearch, 016-030-084). The chemiluminescence was developed using ECL substrate for enhanced chemiluminescence (Thermo Scientific, 32106) and the signal was captured by Chemidoc imaging system (Bio-Rad). Quantification of the bands was performed by Image Lab Software (Bio-Rad).

**Respirometry analysis**

Mitochondrial OCR in HEK293 cells was determined using Seahorse XF96 Extracellular Flux Analyzer (Agilent). Cells were seeded at density of 20 000 cells/well on 0.1mg / mL poly-D-lysine (Merck Millipore) coated XF96 Seahorse plate the day before the experiment. Next day, the medium was replaced 1 h before the measurement with Seahorse XF Base Medium (Agilent #103335–100) supplemented with 1 mM pyruvate, 2 mM glutamine, 25 mM glucose and 5 mM HEPES and pH adjusted to 7.4. The OCR was measured three times in baseline conditions and three times after each injection: 1.5 μM oligomycin (Sigma), 0.25 μM uncoupler carbonilcyanide p-triflouromethoxyphenylhydrazone (FCCP) (Sigma) and 1 μM rotenone (Sigma) and antimycin-A (Sigma). OCR data were normalized to DNA concentration using CyQUANT kit (Invitrogen). Only wells with a homogeneous monolayer of cells were included in the analysis.

**BioID analysis**

143B cells were grown on 15 cm dishes. The cells were transfected with corresponding BirA* fusion constructs using jetPRIME (Polyplus, 114-15) according to the manufacturer's manual. Following 24 h of transfection, 50 μM biotin (Sigma, B4639-1G) was added to each plate and allowed to biotinylate the proximal proteins for the next 24 h. The plates were washed with PBS, and cells scraped, pelleted, and frozen at -80°C. For each sample, four biological replicates were analyzed. Biotinylated proteins were extracted using streptavidin beads (Strep-Tactin sepharose resin, IBA, 2-1201-002) and analyzed by mass spectrometry as previously described (10). The mass spec analysis was done in technical duplicates. The mass spectrometry data were analyzed as previously described (10, 11).

**Co-immunoprecipitation**

One day prior to the transfection, HEK293 CTRL and Full-KO cells were plated on 15 cm dishes. The cells were co-transfected with PTPMT1-V5 and NERCLIN-Strep-HA or with PTPMT1-Strep-HA and NERCLIN-V5 using jetPRIME (Polyplus, 114-15) by 15 μg of each plasmid (30 μg in total). After 24 h mitochondrial isolation was performed followed by immunoprecipitation of the HA-tagged proteins. Briefly, the mitochondria were resuspended in 200 μL of HENN buffer (50 mM HEPES pH 8. 5 mM EDTA, 150 mM NaCl, 50 mM NaF, 0.5% Np40, 1 mM DTT, 1 mM PMSF (Sigma, 93482), 1.5 mM $Na_3VO_4$ and protease inhibitor cocktail (Thermo Scientific, 78444)) and incubated for 20 min on ice. Meanwhile, monoclonal anti-HA−agarose beads produced in mouse (Sigma, A2095) were washed with HENN buffer and centrifuged for 1 min at 4 000 g at 4°C. Lysed mitochondria were centrifuged for 20 min at 14 000 g at 4°C, and 175 µL of the supernatant were added to the washed beads. The remaining 25 µL were used later as input. Samples were incubated on a rotating wheel for 6 h at 4°C and then were centrifuged at 4 000 g for 2 min 4°C. The resulting supernatant (flow-through) was collected into a new tube and the beads were washed three times with 500 µL of HENN buffer and centrifuged at 4 000g for 2 min 4°C. After the final wash, 20 µL of 4 x loading buffer and 20 µL of HENN buffer were added, and samples were boiled for 10 min at 95°C. Equal volumes of each sample were separated by SDS-PAGE followed by western blotting (described in 'Western blotting').

**Immunoprecipitation**

Three replicates were used to prepare samples for IP-MS analysis. 143B cells were plated on 10 cm dishes one day before the transfection. The cells were transfected with NERCLIN-Strep-HA or GFP-Strep-HA using Transit-X2 transfection reagent (Mirus, MIR6000). After 24 h the cells were lysed using HENN buffer (50 mM HEPES pH 8. 5 mM EDTA, 150 mM NaCl, 50 mM NaF, 0.5% Np40, 1 mM DTT, 1 mM PMSF (Sigma, 93482), 1.5 mM Na3VO4 and protease inhibitor cocktail (Thermo Scientific, 78444)) and incubated for 20 min on ice. The protein lysates were centrifuged at for 20 min at 14 000 g at 4°C. Monoclonal anti-HA−agarose beads produced in mouse (Sigma, A2095) or Strep-Tactin sepharose resin (IBA, 2-1201-002) were washed with HENN buffer and centrifuged for 1 min at 4 000 g at 4°C. The clear protein lysates were added to the prewashed beads and incubated on a rotating wheel for 6 h at 4°C. Then the beads were pelleted at 4 000 g for 2 min 4°C and washed three times with HENN buffer. Following immunoprecipitation, the samples were analyzed by mass spectrometry or by immunoblotting. For mass spectrometry analysis the protein complex were eluted from Strep-Tactin beads with the HENN buffer containing biotin (200 mM). The purified protein samples were reduced with 5 mM Tris(2-carboxyethyl)phosphine (TCEP; Sigma-Aldrich) for 20 min at 37°C, and then alkylated with 10 mM iodoacetamide (IAA; Sigma-Aldrich) for 20 min at room temperature in the dark. Sequencing Grade Modified Trypsin (Promega) was then used to get a 1:100 enzyme to substrate ratio and the samples were incubated overnight at 37°C. The samples were desalted with C18 macrospin columns (Nest Group).

For immunoblotting analysis Laemmli sample buffer (Bio–Rad, 1610737) was added onto the washed beads, and the bound complexes were eluted by boiling for 10 min at 95°C, followed by immunoblotting analysis (described in ‘Western blotting’).

**Mass spectrometry analysis of proteins**

The desalted samples were examined with an Evosep One liquid chromatography system connected to a Bruker timsTOF Pro hybrid trapped ion mobility quadrupole TOF mass spectrometer via a CaptiveSpray nano-electrospray ion source. For peptide separation using the 60 samples per day approaches, an 8 cm 150 m column with 1.5 m C18 beads (EV1109, Evosep) was employed (21 min gradient time). The concentrations of formic acid in mobile phases A and B were 0.1% in water and 0.1% in acetonitrile, respectively. The MS analysis was carried out in the positive-ion mode utilizing data-dependent acquisition (DDA) in PASEF mode, with a total of 10 PASEF scans each topN acquisition cycle. MSFragger (12) was used to analyze raw data (.d) obtained in PASEF (13) mode against the human Uniprot database. Only mitochondrially localized proteins were included for further analysis. The mass spectrometry data were filtered based on the FDR (set to 0,05) and the fold change (set to 2).

**Transmission electron microscopy**

HEK293 cells were cultured on glass coverslips in 6-well plates for 24 h, then transfected with the corresponding construct. After 48 h of transfection, the cells were fixed with a solution of 2% glutaraldehyde (Sigma, G7651) in 0.1 M sodium cacodylate buffer at pH 7.4 for 25 min at room temperature. Then the cells were washed two times for 3 min with 0.1 M sodium cacodylate buffer (pH 7.4). Fixed cells were then processed according to the standard protocol at the Electron Microscopy Unit of the Institute of Biotechnology, University of Helsinki. Images were acquired with the Jeol JEM-1400 transmission electron microscope. ImageJ software was used to quantify mitochondrial major axis length (mitochondrial length) or cristae width.

**Mass spectrometric analysis of lipids**

To prepare mitochondrial samples four 15 cm dishes of HEK293 cells were used for each sample. The cells were washed on the plate two times with PBS (Life Technologies, BE17-516F) and once with ice-cold 0.25 M sucrose (Sigma, S0389). Next, the cells were scraped in 0.25 M sucrose and pelleted at 3 000 g for 10 min. The supernatant was removed, and the cell pellets were stored at 80°C. Lipids were extracted from the cell pellets or isolated mitochondria according to the Folch method (14). The samples were analyzed with electrospray ionization-

tandem mass spectrometry (ESI-MS/MS) using 6410 Triple Quadrupole LC/MS (Agilent Technologies, CA, USA). The final lipid extracts in chloroform:methanol (1:2, v:v) were spiked with internal phospholipid standards or Ceramide/Sphingoid Internal Standard Mixture I (Avanti Polar Lipids, USA) and 1% $NH_4OH$ (Merck) right before infusion into the MS at a flow rate of 10 µL/min. The internal phospholipid standards were phosphatidylcholine (PC) 14:1/14:1, PC 20:1/20:1, PC 22:1/22:1; phosphatidylethanolamine (PE) 14:1/14:1 and PE 20:1/20:1; phosphatidylserine (PS) 14:1/14:1; phosphatidylinositol (PI) 16:1/16:1; and sphingomyelin (SM) 18:1/17:0 (from Avanti Polar Lipids or prepared in house). The MS source temperature was 250°C and instrument nitrogen was used as the nebulizing (40 psi) and the drying gas (3 L/min). Precursor ion scans were employed to produce lipid class specific scans as follows: *m/z* 184 for PC and SM; *m/z* 241 for PI; *m/z* 264 for ceramide (Cer) and hexosylceramide (HexCer); and *m/z* 369 for cholesterol ester (CE) as ammonium adducts (15–17). Neutral loss scans were employed for PE (loss of 141 amu) and PS (loss of 87 amu) (15). PE plasmalogens (PEp) were identified with fragment-specific scans for the vinyl ether chains at the *sn*-1 position (e.g., *m/z* 364, 390, and 392 for 16:0p, 18:1p, and 18:0p, respectively) and analyzed from the MS- scan (18). Triacylglycerol (TAG) species were analyzed as ammonium adducts from the MS+ scan (19). For CL and PG species analysis, the lipid extract was spiked with CL 14:0/14:0/14:0/14:0 and PG 20:1/20:1 (Avanti Polar Lipids) internal standards, methylated (20) and the lipids were extracted as above (14). The samples were then run on ACQUITY Ultra Performance LC system coupled to ESI source of Quattro Micro triple quadrupole MS (Waters, Manchester, UK) as described previously (21) and samples of the heat stress experiment were run with 6410 Triple Quadrupole LC/MS (Agilent Technologies). The CL were detected by MS+ scanning, and the PG species were selectively detected by scanning for the neutral loss of 203 amu (22). The spectra were extracted from the chromatogram and all lipid species were identified and quantified using Lipid Mass Spectrum Analysis software (23) and internal and external standards. The lipid species are marked as follows: [lipid class] [sum of acyl chain carbons]:[sum of acyl chain double bonds] (e.g., PC 34:1). The data are described as molar percentages (mol%) for each individual species relative to its lipid class.
The lipidomics data were analyzed with R (https://www.R-project.org/) using limma (24). The raw intensities were normalized with the voom-function. The analysis was done using linear regression (lmFit), with empirical Bayesian statistics (eBayes).

**Fluorometric measurement of total CL**
To measure total CL in cells and isolated mitochondria fluorometric CL assay kit (BioVision, K944) was used. Cells or mitochondrial pellets were resuspended in the CL assay buffer and lysed by sonication. The debris was removed by centrifugation at 10 000 rpm for 10 min at +4°C. Then, the CL measurement was performed according to the manufacturer's instructions.

**Cell viability assay**
ATPlite viability assay kit (PerkinElmer, 6016943) was used to analyze cell viability. HEK293 cells were seeded on 96-well plates two days before the experiment to reach ~90% confluence the day of the experiment. The cells were washed twice with HBSS solution (Gibco, 14025092) and 100 µL of HBSS was added to each well. Then, the cells were exposed for 1 h or 2 h at 45°C in a cell incubator in normoxia and 5% CO2. Control cells were kept at 37°C. All samples were lysed at the same time prior to the assay, which was performed according to the manufacturer's recommendations. Luminescence was monitored with an EnSpire (PerkinElmer) microplate reader with 0.1 s measurement time and normalized to protein levels determined in parallel wells by bicinchoninic acid (BCA) assay (Thermo Fisher, 23227).

**Statistical analysis**
All data are presented as mean ± standard deviation (SD). For the statistical analyses with two samples, unpaired two-tailed t-tests or one-way ANOVA analysis were performed using Graph Pad Prism Software. All the graphs were prepared with Graph Pad Prism Software.

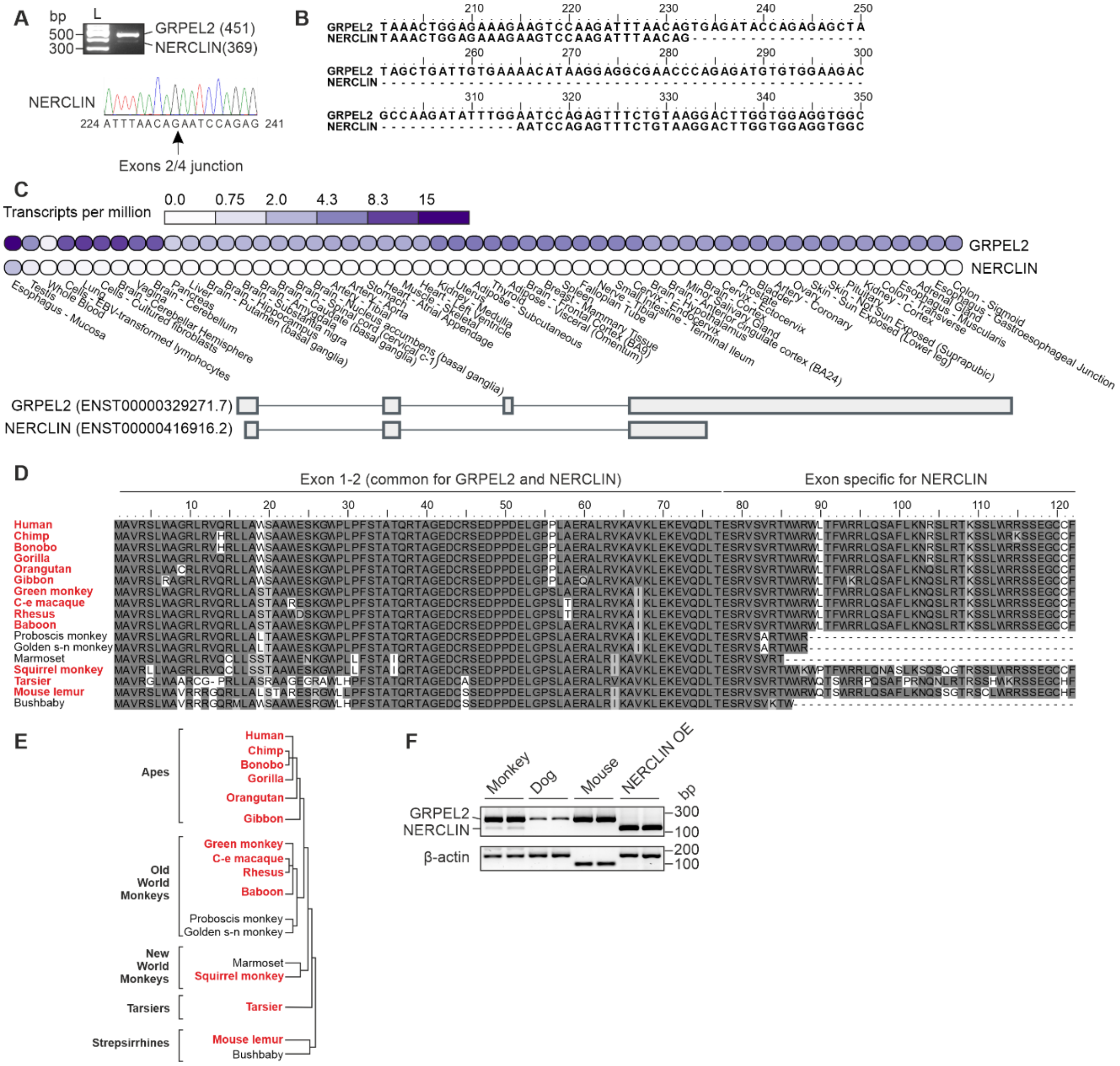


**Fig. S1. Expression and conservation of NERCLIN.** (A) RT-PCR co-amplification of two GRPEL2 transcripts in 143B cells. Primers spanning NERCLIN cDNA were used (upper panel). The RT-PCR products were run on agarose gel. The expected band sizes in bp are indicated in brackets. L, DNA ladder. RT-PCR products were cloned and sequenced. DNA sequencing chromatogram indicating junction of exon 2 and 4 in GRPEL2 variant (lower panel). (B) cDNA sequence alignment of GRPEL2 and NERCLIN lacking exon 3 (233–724 bp). The RT-PCR products as in (A) were used for nucleotide sequencing. (C) Analysis of GRPEL2 isoform expression using Genotype-Tissue Expression (GTEx) portal (https://gtexportal.org/home/). (D) Multiple sequence alignment of GRPEL2 protein transcript skipping exon 3 in primates. Identical residues are highlighted in dark gray, similar residues are in light gray. Species that are predicted to have NERCLIN are in red. C-e macaque, crab-eating macaque; Golden s-n monkey, golden snub-nosed monkey. (E) Phylogeny tree of primates analyzed in (D). Species that are predicted to have NERCLIN are in red. (F) NERCLIN transcript detection in monkey kidney cells (COS7), dog fibroblasts and mouse liver. RT-PCR products of GRPEL2 and NERCLIN, 260 bp and 168 bp respectively were detected by agarose gel electrophoresis. HEK293 cells overexpressing NERCLIN were used as a positive control. B-actin was used as a loading control.

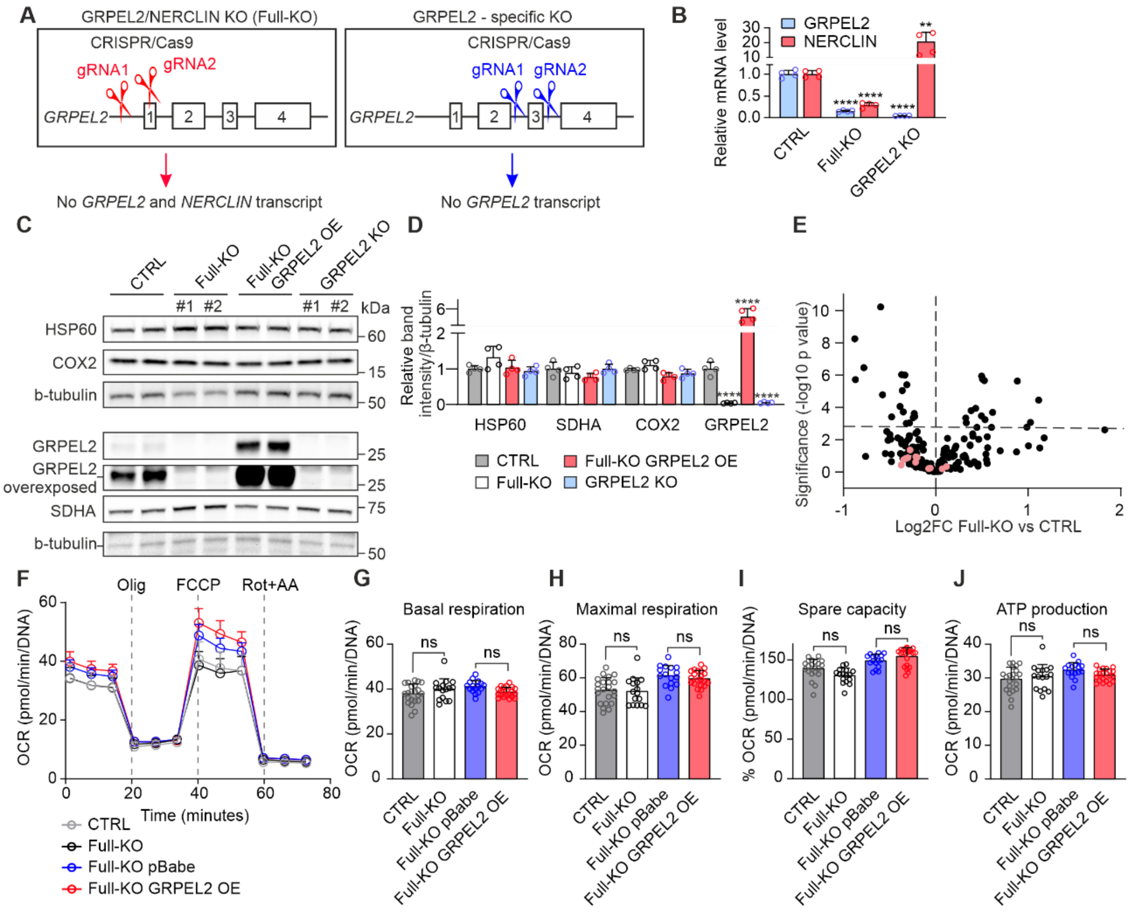


**Fig. S2. GRPEL2/NERCLIN knockout or GRPEL2 knockout does not cause mitochondrial abnormalities or cardiolipin depletion.** (A) CRISPR/Cas9 approach using guide RNAs (gRNAs) targeted to specific regions of the GRPEL2 gene to generate GRPEL2/NERCLIN knockout or GRPEL2 knockout HEK293 cells. (B) GRPEL2 or NERCLIN transcript levels in GRPEL2/NERCLIN knockout or GRPEL2 knockout cells determined by qPCR assay (n = 4). (C) Western blot analysis of mitochondrial proteins in GRPEL2/NERCLIN knockout (Full-KO), Full-KO cells stably overexpressing GRPEL2 (Full-KO GRPEL2 OE) or GRPEL2 knockout cells. #1 and #2 are two independent clonal cell lines. (D) Quantification of western blot images presented in (C). Protein expression levels are normalized to β-tubulin level (n = 4). In (B) and (D) the data are presented as mean ± SD. **$P < 0.01$, ****$P < 0.0001$, ns, not significant as compared to the control cells (unpaired t-tests). (E) Volcano plot comparing the lipid species composition of GRPEL2/NERCLIN knockout (KO) HEK293 cells and control cells (CTRL). The dashed line indicates a false discovery rate (FDR)-corrected P-value of 0.05. CL species are in light red. (F-J) Mitochondrial respiration in cells lacking NERCLIN is not affected. High-resolution respirometry assay using Seahorse Analyzer was performed in GRPEL2/NERCLIN knockout (Full-KO) cells, Full-KO cells stably overexpressing GRPEL2 (Full-KO GRPEL2 OE) or Full-KO cells with stably introduced empty vector (Full-KO pBabe) and control cells (CTRL) (n = 16 – 22 measurements per cell line). The data are presented as mean ± SD. ns, not significant (one-way ANOVA).

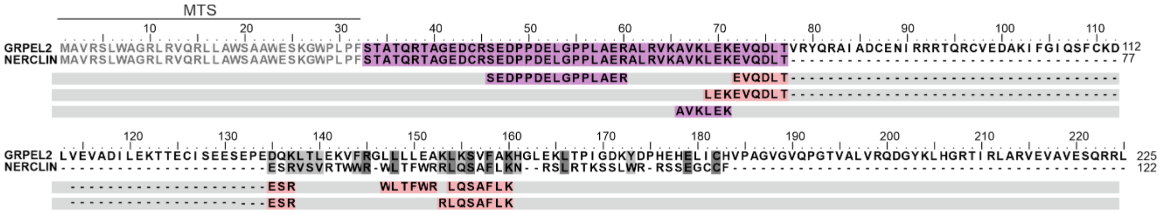

**Fig. S3. NERCLIN does not show proximity to GRPEL2.** Peptide spectrum matches of GRPEL2 in 143B cells overexpressing NERCLIN-BirA* identified by mass spectrometry in BioID analysis. Amino acid sequence alignment of human GRPEL2 and NERCLIN. Identical residues are highlighted in dark gray, similar residues are in light gray. Amino acid sequence common for GRPEL2 and NERCLIN is in violet. Peptides unique to NERCLIN identified in cells overexpressing NERCLIN-BirA* are in light red. Mitochondrial targeting sequence, MTS (1-32 amino acids) is in gray.

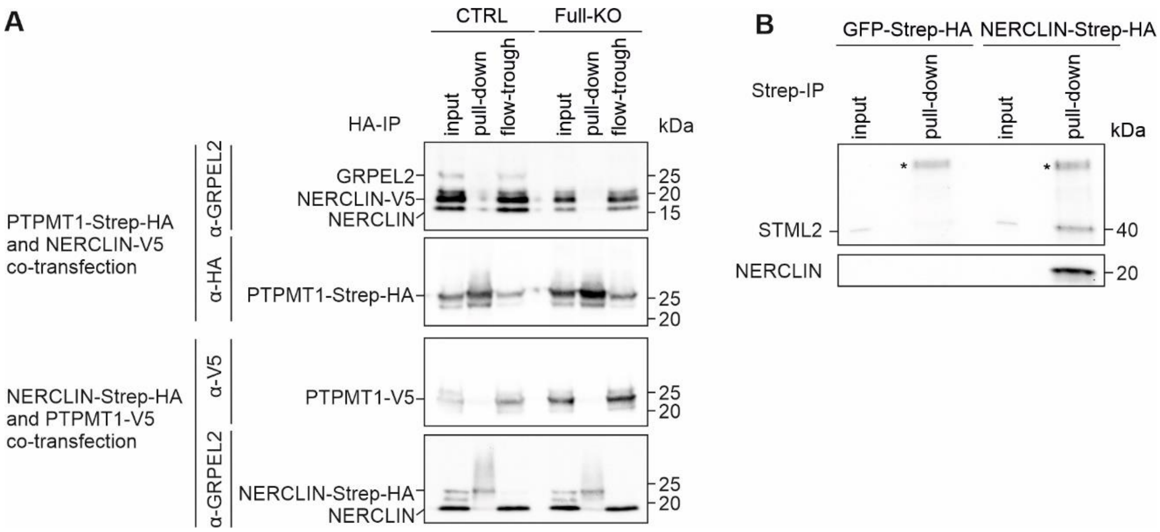


**Fig. S4. Immunoprecipitation analysis shows direct interaction between NERCLIN and STML2.** (A) Control HEK293 cells or cells lacking GRPEL2 and NERCLIN (Full-KO) were co-transfected with PTPMT1-Strep-HA and NERCLIN-V5 or with NERCLIN-Strep-HA and PTPMT1-V5 for 24 h. Isolated mitochondria were used for immunoprecipitation using anti-HA agarose beads (HA-IP). The proteins were detected by western blot. (B) 143B cells were transfected with GFP-Strep-HA or with NERCLIN-Strep-HA. Immunoprecipitation was performed using Strep-Tactin Sepharose beads (Strep-HA). The proteins were analyzed by western blot. Stars represent non-specific bands.

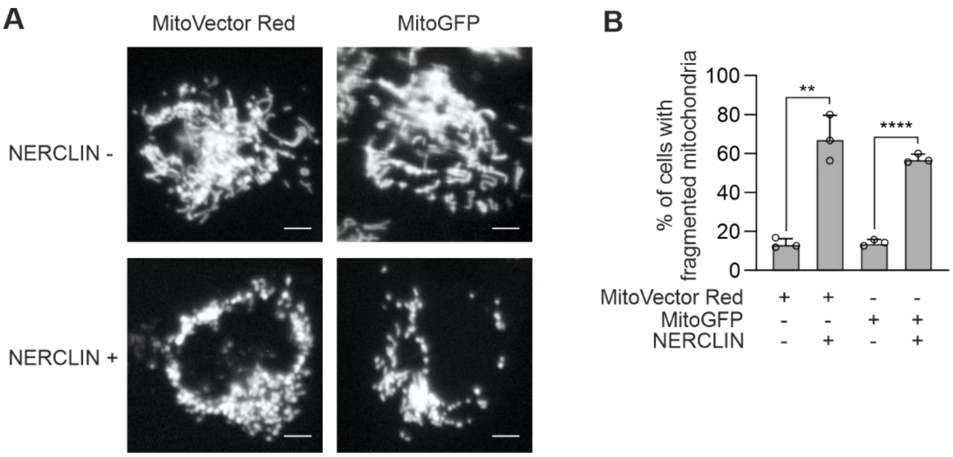


**Fig. S5. NERCLIN overexpression causes mitochondrial fragmentation in live cells.** 143B cells were transiently transfected with MitoVector Red or MitoGFP alone or together with NERCLIN. The cells were analyzed after 24 h of transfection. (A) Live microscopy images showing mitochondrial morphology. MitoVector Red and MitoGFP were visualized using red channel and green channel respectively. Scale bar, 5 μm. (B) Quantification analysis of cells with fragmented mitochondria. At least 50 cells for each condition were analyzed. Data are presented as mean ± SD. **P < 0.01, ****P < 0.0001, as compared to the cells transfected with MitoVector Red or MitoGFP alone (unpaired t-tests).

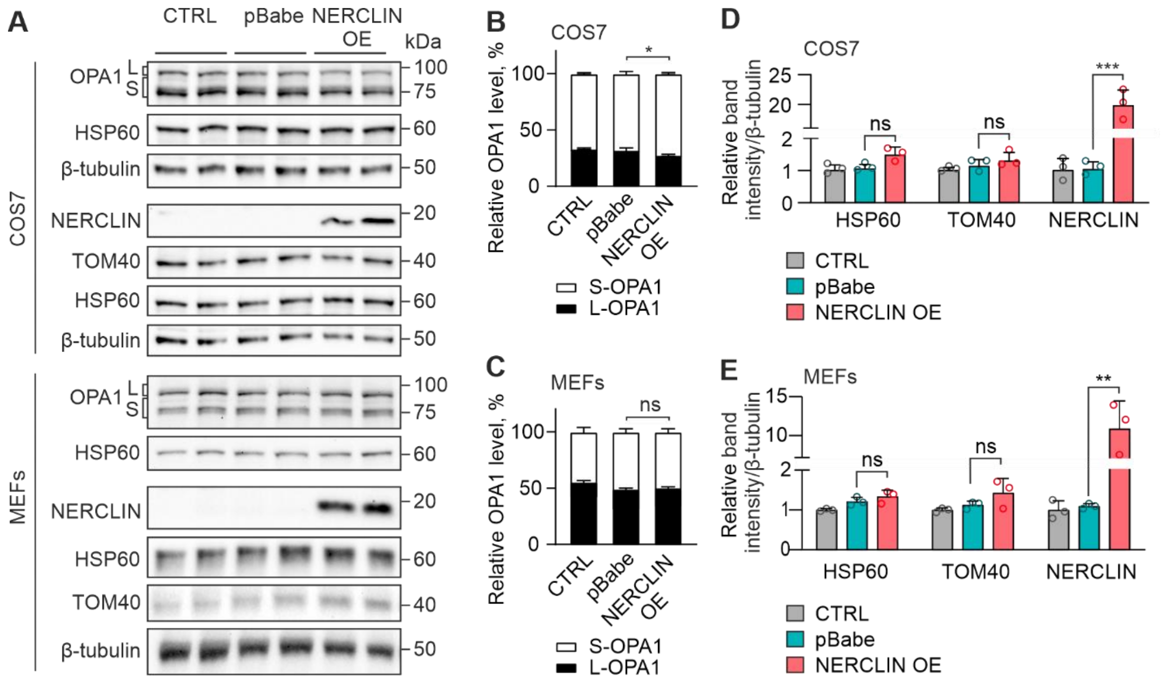


**Fig. S6. Overexpression of NERCLIN specifically affects mitochondria in primate cells.** (A) Western blot analysis of African green monkey kidney fibroblasts (COS7) or mouse embryonic fibroblasts (MEFs) overexpressing NERCLIN. Cells were transiently transfected with NERCLIN plasmid (NERCLIN OE) or with an empty vector (pBabe) for 48 h. CTRL, non-transfected cells. (B, C) Relative levels of short and long OPA1 isoforms determined by western blot analysis in (A). Total OPA1 level was taken as 100%. L, long OPA1 isoform, S, short OPA1 isoform (n = 3). (D, E) Quantification of western blot images presented in (A). Protein expression levels are normalized to β-tubulin level (n = 3). In all graphs data are presented as mean ± SD. *P < 0.05, **P < 0.01, ***P < 0.001, ns, not significant as compared to the cells transfected with empty vector (unpaired t-tests).

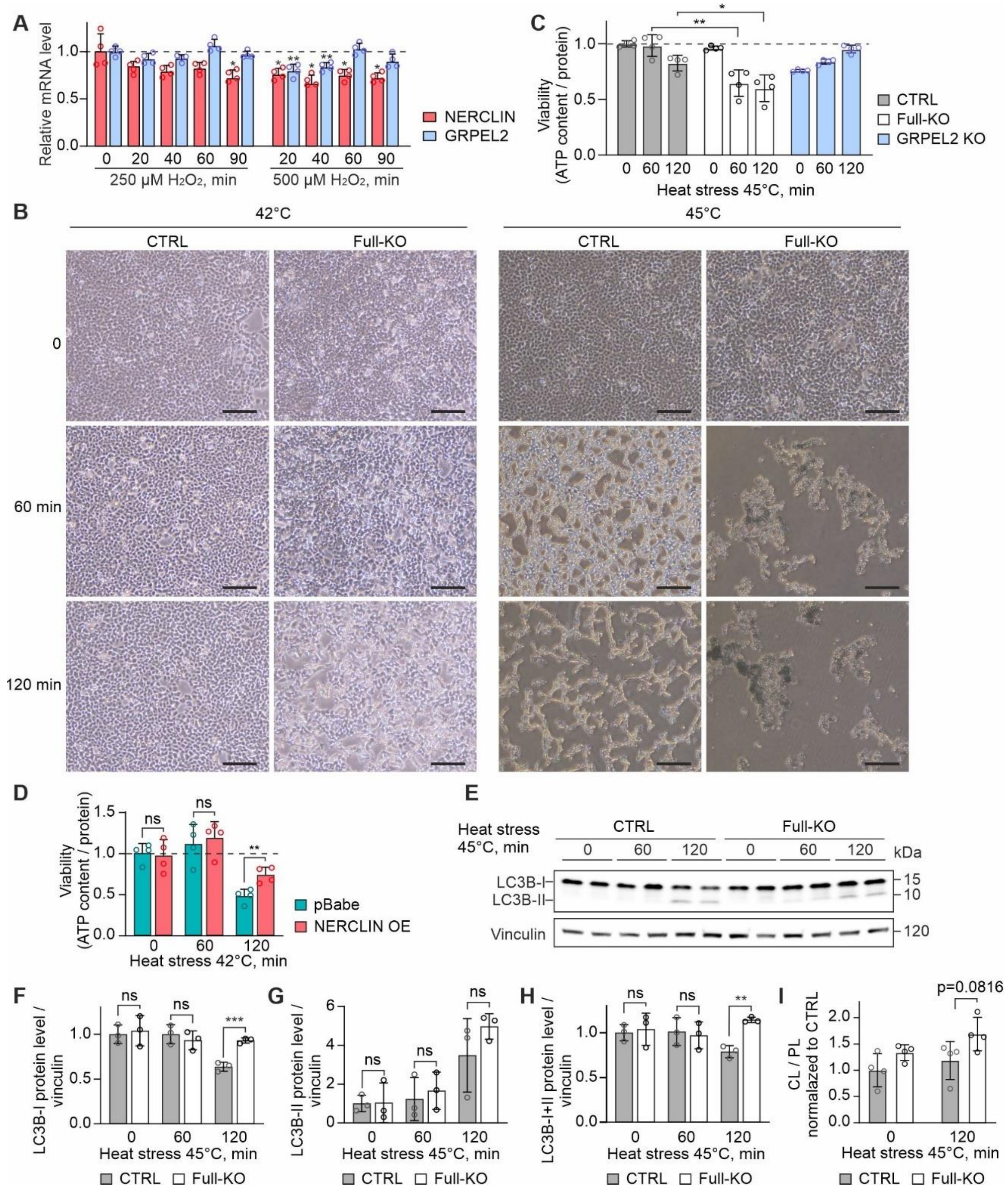


**Fig. S7. NERCLIN protects cells from heat stress.** (A) mRNA levels of NERCLIN or GRPEL2 in HEK293 cells exposed to $H_2O_2$ as determined by qPCR assay (n = 4). (B) Live microscopy images of HEK293 control cells (CTRL) or cells lacking GRPEL2 and NERCLIN (Full-KO) exposed to heat stress (42°C or 45°C). Scale bar: 200 µm. (C) Viability of CTRL, Full-KO or GRPEL2 KO HEK293 cells exposed to heat stress (45°C). ATPlite viability assay kit was used to analyze cell viability. ATP content was normalized to protein levels. (D) Viability of HEK293 control cells transiently transfected for 24 h with NERCLIN plasmid (NERCLIN OE) or with an empty vector (pBabe) exposed to heat stress (42°C). ATPlite viability assay kit was used to analyze cell viability. ATP content was normalized to protein levels. The viability relative to the

untreated cells transfected with empty vector is shown. (E) Western blot analysis of HEK293 control cells (CTRL) or cells lacking GRPEL2 and NERCLIN (Full-KO) exposed to heat stress (45°C). Vinculin was used as a loading control. (F-H) Relative levels of LC3B isoforms determined by western blot analysis in (E) (n = 3). (I) CL concentrations were analyzed by mass spectrometry lipidomics in CTRL or Full-KO cells before and after 2 h of heat stress (45°C). CL concentrations related to the total concentrations of other measured phospholipids (phosphatidylcholines, phosphatidylcholine alkyls, phosphatidylethanolamines, phosphatidylinositols and sphingomyelins) are presented (n = 4). In all graphs data are presented as mean ± SD. *P < 0.05, **P < 0.01, ***P < 0.001, ns, not significant as compared to untreated CTRL cells (unpaired t-tests).

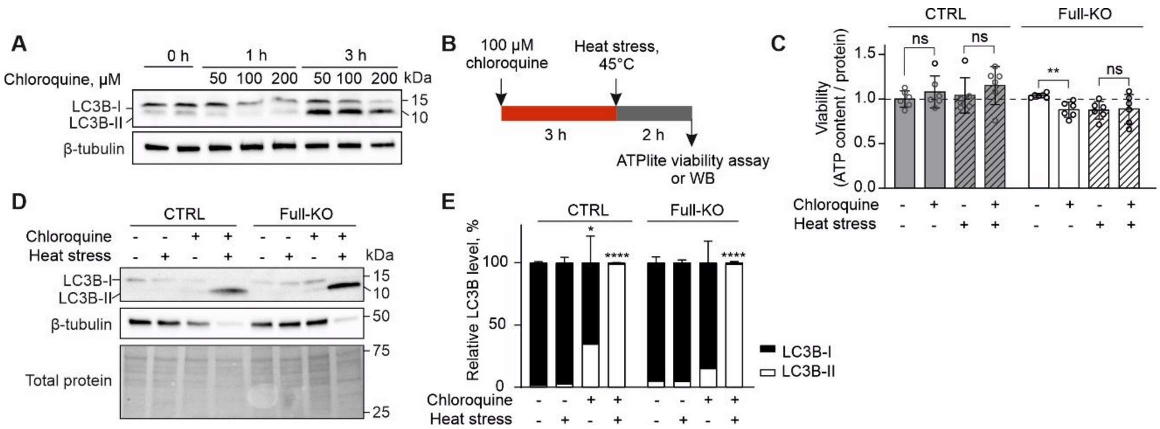


**Fig. S8. Mitophagy does not contribute to the heat-induced cell death.** (A) Chloroquine efficiently inhibits autophagic flux and induces LC3B-II accumulation as assessed by western blotting. (B) Experimental design of ATPlite viability assay and western blotting of the cells treated with the mitophagy inhibitor, chloroquine, followed by heat stress. (C) Viability of CTRL or cells lacking GRPEL2 and NERCLIN (Full-KO) HEK293 cells treated as shown in (B). Viability was measured by ATPlite viability assay kit and normalized to protein levels. (D) Western blot analysis of CTRL or Full-KO HEK293 cells treated as shown in (B). (E) Relative levels of LC3B isoforms as determined by western blotting (D) (n = 3). In all graphs data are presented as mean ± SD. *P < 0.05, **P < 0.01, ****P < 0.0001, ns, not significant (unpaired t-tests).

**Dataset S1 (separate file).** High confidence interactors of NERCLIN identified by BioID approach.

**Dataset S2 (separate file).** NERCLIN interactors identified by IP-MS.

**Dataset S3 (separate file).** Lipidomics analysis of cells overexpressing NERCLIN.

**Dataset S4 (separate file).** Lipidomics analysis of cells exposed to heat stress.

**Dataset S5 (separate file).** List of primers used in this study.

**Dataset S6 (separate file).** Source data.

## SI References


1. A. Drozdetskiy, C. Cole, J. Procter, G. J. Barton, JPred4: a protein secondary structure prediction server. *Nucleic Acids Res* **43**, W389-94 (2015).
2. C. Combet, C. Blanchet, C. Geourjon, G. Deleage, NPS@: network protein sequence analysis. *Trends Biochem Sci* **25**, 147–150 (2000).
3. D. W. A. Buchan, D. T. Jones, The PSIPRED Protein Analysis Workbench: 20 years on. *Nucleic Acids Res* **47**, W402–W407 (2019).
4. W. J. Kent, *et al.*, The human genome browser at UCSC. *Genome Res* **12**, 996–1006 (2002).
5. R. Trokovic, J. Weltner, T. Otonkoski, Generation of iPSC line HEL24.3 from human neonatal foreskin fibroblasts. *Stem Cell Res* **15**, 266–268 (2015).
6. X. Liu, *et al.*, An AP-MS- and BioID-compatible MAC-tag enables comprehensive mapping of protein interactions and subcellular localizations. *Nat Commun* **9**, 1188-018-03523–2 (2018).
7. C. Frezza, S. Cipolat, L. Scorrano, Organelle isolation: functional mitochondria from mouse liver, muscle and cultured fibroblasts. *Nat Protoc* **2**, 287–295 (2007).
8. R. Torregrosa-Munumer, *et al.*, PrimPol is required for replication reinitiation after mtDNA damage. *Proc Natl Acad Sci U S A* **114**, 11398–11403 (2017).
9. J. Saarimaki-Vire, *et al.*, An Activating STAT3 Mutation Causes Neonatal Diabetes through Premature Induction of Pancreatic Differentiation. *Cell Rep* **19**, 281–294 (2017).
10. S. Konovalova, *et al.*, Redox regulation of GRPEL2 nucleotide exchange factor for mitochondrial HSP70 chaperone. *Redox Biol* **19**, 37–45 (2018).
11. X. Liu, K. Salokas, R. G. Weldatsadik, L. Gawriyski, M. Varjosalo, Combined proximity labeling and affinity purification-mass spectrometry workflow for mapping and visualizing protein interaction networks. *Nat Protoc* (2020).
12. F. Yu, *et al.*, Fast Quantitative Analysis of timsTOF PASEF Data with MSFragger and IonQuant. *Mol Cell Proteomics* **19**, 1575 (2020).
13. F. Meier, *et al.*, Online Parallel Accumulation–Serial Fragmentation (PASEF) with a Novel Trapped Ion Mobility Mass Spectrometer. *Mol Cell Proteomics* **17**, 2534 (2018).
14. J. FOLCH, M. LEES, G. H. S. STANLEY, A simple method for the isolation and purification of total lipides from animal tissues. *J Biol Chem* **226**, 497–509 (1957).

15. B. Brügger, G. Erben, R. Sandhoff, F. T. Wieland, W. D. Lehmann, Quantitative analysis of biological membrane lipids at the low picomole level by nano-electrospray ionization tandem mass spectrometry. *Proceedings of the National Academy of Sciences* **94**, 2339–2344 (1997).
16. G. Liebisch, *et al.*, Quantitative measurement of different ceramide species from crude cellular extracts by electrospray ionization tandem mass spectrometry (ESI-MS/MS). *J Lipid Res* **40**, 1539–1546 (1999).
17. K. Duffin, M. Obukowicz, A. Raz, J. J. Shieh, Electrospray/tandem mass spectrometry for quantitative analysis of lipid remodeling in essential fatty acid deficient mice. *Anal Biochem* **279**, 179–188 (2000).
18. K. A. Z. Berry, R. C. Murphy, Electrospray ionization tandem mass spectrometry of glycerophosphoethanolamine plasmalogen phospholipids. *J Am Soc Mass Spectrom* **15**, 1499–1508 (2004).
19. K. L. Duffin, J. D. Henion, J. J. Shieh, Electrospray and tandem mass spectrometric characterization of acylglycerol mixtures that are dissolved in nonpolar solvents. *Anal Chem* **63**, 1781–1788 (1991).
20. J. Clark, *et al.*, Quantification of PtdInsP3 molecular species in cells and tissues by mass spectrometry. *Nat Methods* **8**, 267–272 (2011).
21. V. Kainu, M. Hermansson, S. Hanninen, K. Hokynar, P. Somerharju, Import of phosphatidylserine to and export of phosphatidylethanolamine molecular species from mitochondria. *Biochim Biophys Acta* **1831**, 429–437 (2013).
22. X. Wang, *et al.*, Quantitative molecular tissue atlas of Bis(monoacylglycero)phosphate and phosphatidylglycerol membrane lipids in rodent organs generated by methylation assisted high resolution mass spectrometry. *Anal Chim Acta* **1084**, 60–70 (2019).
23. P. Haimi, A. Uphoff, M. Hermansson, P. Somerharju, Software tools for analysis of mass spectrometric lipidome data. *Anal Chem* **78**, 8324–8331 (2006).
24. M. E. Ritchie, *et al.*, limma powers differential expression analyses for RNA-sequencing and microarray studies. *Nucleic Acids Res* **43**, e47 (2015).
